# Mechanical nanosurgery of chemoresistant glioblastoma using magnetically controlled carbon nanotubes

**DOI:** 10.1126/sciadv.ade5321

**Published:** 2023-03-29

**Authors:** Xian Wang, Zheyuan Gong, Tiancong Wang, Junhui Law, Xin Chen, Siyi Wanggou, Jintian Wang, Binbin Ying, Michelle Francisco, Weifan Dong, Yi Xiong, Jerry J. Fan, Graham MacLeod, Stephane Angers, Xuejun Li, Peter B. Dirks, Xinyu Liu, Xi Huang, Yu Sun

**Affiliations:** ^1^ Program in Developmental and Stem Cell Biology, The Hospital for Sick Children, Toronto, ON, Canada.; ^2^ Arthur and Sonia Labatt Brain Tumour Research Centre, The Hospital for Sick Children, Toronto, ON, Canada.; ^3^ Department of Mechanical and Industrial Engineering, University of Toronto, Toronto, ON, Canada.; ^4^ Songjiang Hospital, Shanghai Jiao Tong University School of Medicine, Shanghai 200025, China.; ^5^ Department of Neurosurgery, Xiangya Hospital, Central South University, Changsha, Hunan, China.; ^6^ Hunan International Scientific and Technological Cooperation Base of Brain Tumor Research, Xiangya Hospital, Central South University, Changsha, Hunan, China.; ^7^ Department of Molecular Genetics, Temerty Faculty of Medicine, University of Toronto, Toronto, ON, Canada.; ^8^ Donnelly Centre for Cellular & Biomolecular Research, University of Toronto, Toronto, ON, Canada.; ^9^ Leslie Dan Faculty of Pharmacy, University of Toronto, Toronto, ON, Canada.; ^10^ Department of Biochemistry, Temerty Faculty of Medicine, University of Toronto, Toronto, ON, Canada.; ^11^ Institute of Biomedical Engineering, University of Toronto, Toronto, ON, Canada.; ^12^ Department of Electrical and Computer Engineering, University of Toronto, Toronto, ON, Canada.; ^13^ Department of Computer Science, University of Toronto, Toronto, ON, Canada.

## Abstract

Glioblastoma (GBM) is the most common and aggressive primary brain cancer. Despite multimodal treatment including surgery, radiotherapy, and chemotherapy, median patient survival has remained at ~15 months for decades. This situation demands an outside-the-box treatment approach. Using magnetic carbon nanotubes (mCNTs) and precision magnetic field control, we report a mechanical approach to treat chemoresistant GBM. We show that GBM cells internalize mCNTs, the mobilization of which by rotating magnetic field results in cell death. Spatiotemporally controlled mobilization of intratumorally delivered mCNTs suppresses GBM growth in vivo. Functionalization of mCNTs with anti-CD44 antibody, which recognizes GBM cell surface–enriched antigen CD44, increases mCNT recognition of cancer cells, prolongs mCNT enrichment within the tumor, and enhances therapeutic efficacy. Using mouse models of GBM with upfront or therapy-induced resistance to temozolomide, we show that mCNT treatment is effective in treating chemoresistant GBM. Together, we establish mCNT-based mechanical nanosurgery as a treatment option for GBM.

## Teaser

Magnetic nanomaterials under field control can function as scalpels to treat therapy-resistant brain tumors.

## INTRODUCTION

Glioblastoma (GBM) is the deadliest brain cancer. The incidence of GBM ranges from 0.59 to 5 per 100,000 people and is on the rise globally (*1*). Standard treatment, which includes maximal safe surgical resection, followed by radiotherapy and chemotherapy, yields a median patient survival of ~15 months (*2*), which has not changed over the past two decades. The chemotherapy drug temozolomide (TMZ), which received U.S. Food and Drug Administration (FDA) approval in 2005, extended patient survival for ~2 months (between patients who received TMZ + radiotherapy and patients who received radiotherapy alone) (*3*–*5*). GBM universally develops resistance to TMZ and other therapeutic agents that target molecules within biochemical pathways, which leads to treatment failure, tumor relapse, and patient mortality (*6*, *7*). Therefore, an approach that can effectively treat chemoresistant GBM is urgently needed but currently unavailable.

Nanomedicine uses nanomaterials [e.g., carbon nanotubes (CNTs), nanoparticles, and nanodiscs] or organic nanostructures (e.g., DNA origami and liposomes) for drug delivery (*8*–*10*), medical imaging (*11*–*14*), and tissue regeneration (*15*). Nanomaterials offer therapeutic efficacy through their tissue permeation, interaction with an external energy source, and capability to be combined with other therapeutic modalities (*16*, *17*). Because we recently demonstrated that GBM cells are mechanosensitive (*18*), we set to use nanomaterials to develop a nanoscale mechanical approach to treat GBM. Mechanical perturbation has been investigated as an approach to target cancer cells. For example, magnetic field–actuated nanomaterials compromise the integrity of plasma membrane, leading to the death of in vitro–cultured GBM cells (*19*) and breast cancer cells (*20*). GBM cells, which were preincubated with magnetic nanoparticles, were implanted into mice to generate xenograft tumors. A rotating magnetic field, which was then applied to these magnetic particles–harboring tumors, suppressed GBM growth (*21*). Similarly, magnetic field mobilization of mitochondria-targeting magnetic nanoparticle chains demonstrated efficacy in inhibiting GBM growth in mice (*22*). While these studies showed that magnetic field–controlled nanomaterials can be used in cancer treatment, the utility of magnetic nanomaterials in treating chemoresistant tumors, the root cause of tumor relapse and patient death, remains unexplored.

GBM displays an extreme level of heterogeneity at genomic, epigenetic, biochemical signaling, and cellular composition levels (*23*). The heterogeneous nature of GBM confers treatment resilience to tumors and leads to a unifying therapy resistance mechanism; i.e., suppressing selected proteins or biochemical pathways provides a fertile ground for alternative signaling mechanisms, which are not targeted by the given therapy, to fuel GBM growth (*24*). In other words, the “whack-a-mole” approach failed to benefit patients with GBM for decades. For this reason, we hypothesized that nanomaterial-based mechanical treatment of cancer cells, rather than specific targeting of signaling pathways, can overcome the therapy resistance of this biologically plastic disease. To this end, we engineered a mechanical nanosurgery approach using magnetic CNTs (mCNTs; nanotubes with carbon surface and a cavity filled with iron particles) based on the following reasons. First, mCNTs display biocompatibility, as their carbon surface serves as a barrier to prevent contact between cells and iron inside the mCNTs (*25*). Second, the magnetic material (iron) enables mCNTs to render mechanical work to tumor cells under a magnetic field. Third, the carbon surface of mCNTs provides a platform for antibody functionalization, which can increase mCNT distribution in tumors through the recognition of tumor cell surface antigens. Here, we show that a spatiotemporally controlled rotating magnetic field activates mCNTs to generate mechanical work and induce GBM cell death in vitro and in vivo. We demonstrate that mCNT surface functionalization using CD44 antibody enhances its tumor tissue enrichment and retention, thereby increasing efficacy in treating GBM in mice. Furthermore, we establish that the mCNT approach is effective in treating GBM with upfront or therapy-induced resistance to TMZ. Collectively, we report a mechanical nanosurgery approach to treat chemoresistant GBM.

## RESULTS

### Effects of mCNTs on GBM cell death under rotating magnetic field

**Fig. 1.**
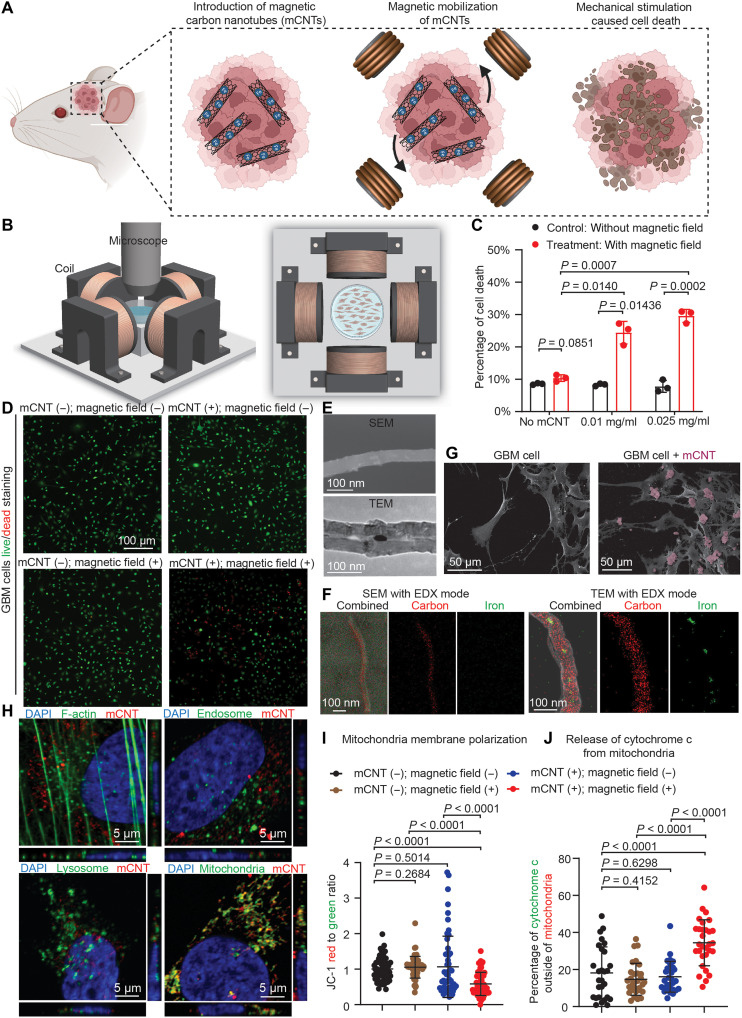
mCNTs induce cell death under rotating magnetic field. (**A**) Rotating magnetic field and magnetic carbon nanotubes (mCNTs) exert mechanical work and stimulation to tumor cells. (**B**) Magnetic coil system for generating rotating magnetic fields. (**C**) Cell death rate after magnetic treatment using different concentrations of mCNTs. Control group receives phosphate-buffered saline (PBS) without mCNTs. *n* = 3 independent experiments. Error bar: SD. (**D**) Representative images of live/dead cell staining of control, mCNT treatment, magnetic treatment, or mCNT + magnetic treatment group. (**E**) Scanning electron microscopy (SEM) and transmission EM (TEM) image of an mCNT. (**F**) SEM and TEM image of an mCNT with energy-dispersive x-ray spectroscopy (EDX) mode. (**G**) SEM images of glioblastoma (GBM) cells with mCNTs. mCNT clusters were labeled with pseudocolor. (**H**) mCNTs and their localization with F-actin, endosome, lysosome, and mitochondria. DAPI, 4′,6-diamidino-2-phenylindole. (**I**) Quantification of mitochondria membrane potential by JC-1 dye staining. JC-1 red to green ratio is calculated by dividing red over green fluorescent signal for each cell. *n* = 50 cells. Error bar: SD. (**J**) Quantification of cytochrome c signal outside of mitochondria. Mitochondria are labeled by TOM20. The percentage of cytochrome c outside of mitochondria is calculated by dividing the cytochrome c signal, which is not overlapped with TOM20, and the total cytochrome c signal for each cell. *n* = 30 cells. Error bar: SD.

For mCNTs, magnetic materials such as iron particles can be incorporated either on the surface or inside the nanotubes. We determined that mCNTs with iron particles filled inside caused less cytotoxicity (fig. S1 and section S1 of the Supplementary Materials), consistent with the fact that the carbon surface of mCNTs is a barrier that prevents contact between iron and cells. The iron-filled mCNTs used in this study have an outer diameter of 70.77 ± 7.24 nm and an inner diameter of 25.19 ± 6.31 nm [*n* = 30 mCNTs under transmission electron microscopy (TEM), error bar: SD], with an iron percentage of 52 weight % (detailed fabrication, size, and magnetic properties described in Methods). We first cultured GBM stem cells (G411) with mCNTs of varying concentrations (0.01, 0.025, and 0.05 mg/ml) for 24 hours. Next, we placed the petri dish in a custom-built magnetic field generation system (Fig. 1B). A uniform rotating magnetic field was applied with a field strength of 20 ± 2 mT and a rotating frequency of 20 Hz (fig. S1A and movie S1), under which the mCNTs tended to align with the direction of the magnetic field (i.e., the direction of magnetic flux density B), inducing mechanical work to stimulate cellular structures. Details on magnetic field parameters are described in section S2 of the Supplementary Materials. The cells underwent magnetic field treatment for 30 min, and the live/dead cell ratio was measured (Fig. 1, C and D). The results showed that mCNT + magnetic field treatment induced marked cell death (e.g., ~30% cell death rate at mCNT concentration of 0.025 mg/ml). Of note, the viability of cells under a uniform rotating magnetic field without mCNTs was not affected compared to the control (*P* = 0.0851; Fig. 1C). Because mCNT concentration of 0.025 mg/ml did not result in toxicity to cells without the application of magnetic field (*P* = 0.5534; fig. S2), we used this mCNT concentration in subsequent experiments.

The effect of time (0, 6, 12, 24, and 48 hours) was studied with mCNT concentration of 0.025 mg/ml under consistent magnetic field parameters (field strength: 20 ± 2 mT and rotating frequency: 20 Hz). We found that the internalization rate, defined as mCNT area over the cell spreading area under bright field microscopy, plateaued between 6 and 12 hours (fig. S3A). Correspondingly, the cell death rate reached a plateau between 6 and 12 hours. No difference in internalization or cell death rate was observed among 12, 24, and 48 hours after cell seeding (fig. S3B). We tested the effectiveness of mCNT + magnetic field treatment using additional patient-derived GBM cell lines. In all GBM cell lines, mCNT + magnetic field treatment significantly elevated cell death rates compared to mCNT without magnetic field (fig. S4). We noted that the cell death rate caused by the mCNT + magnetic field treatment differs among different cell types, likely because of the different amounts of mCNTs internalized. A cell’s capability of internalizing mCNTs can depend on several factors, such as adhesion proteins on the cell surface (*26*), cell membrane dynamics (*27*), and adenosine 5′-triphosphate consumption (*28*).

### mCNT distribution in GBM cells

We next performed scanning electron microscopy (SEM) and TEM imaging to assess the mCNTs and determine mCNT distribution in relationship with GBM cells (Fig. 1, E and F). Iron particles were observed inside mCNTs under TEM but not on their surface under SEM (Fig. 1E). SEM and TEM with energy-dispersive x-ray spectroscopy (EDX) showed mCNTs with carbon surface and iron inside (Fig. 1F). The corresponding EDX spectrum of mCNTs showed peaks of iron and carbon, with no peaks other than carbon, iron, oxygen, or nitrogen observed (fig. S5). To investigate mCNT distribution in relationship with GBM cells, mCNTs were supplied to cell culture media for 24 hours. GBM cells were then washed extensively with phosphate-buffered saline (PBS) and fixed for EM and fluorescence imaging. SEM showed the presence of mCNTs on GBM cell surface (Fig. 1G). The majority of mCNTs (63.48 ± 25.89%) were present in the cytoplasm of GBM cells as clusters with an average cluster length of 0.48 μm (fig. S6).

To investigate the entry route of mCNTs into GBM cells, we used endocytosis inhibitors (Dynasore and Pitstop to inhibit clathrin-mediated endocytosis and Nysterin to inhibit caveolin-mediated endocytosis) and determined their effects on mCNT internalization (*29*, *30*). GBM cells were treated with vehicle or endocytosis inhibitors and cultured with mCNT for 24 hours. Internalization was quantified by dividing mCNT area over the total cell spreading area in each cell (i.e., mCNT localization ratio). We found that mCNT localization ratio significantly decreased after treating cells with inhibitors of either clathrin- or caveolin-mediated endocytosis, demonstrating that endocytosis is an essential route through which mCNTs enter GBM cells (fig. S7A).

To further investigate mCNT localization within GBM cell, we performed TEM imaging and identified mCNTs at endosome, lysosome, and mitochondria after culturing GBM cells with mCNTs for 24 hours (fig. S7B). Furthermore, we tagged mCNTs with fluorophore and cultured mCNTs with cells for 2, 6, 12, or 24 hours (Fig. 1H). Consistent with our observations using TEM, mCNTs colocalized with markers of endosome and lysosome. Over the culturing periods (fig. S7C), we detected a decrease of mCNT localization at endosome and lysosome (at 24 hours: 9.11 ± 3.20% mCNT localization at endosome and 4.921 ± 0.488% mCNT localization at lysosome) and an increase of mCNT localization at mitochondria (at 24 hours: 31.68 ± 4.23 mCNT localization at mitochondria; Fig. 1H). While this dynamic mCNT subcellular localization warrants future investigation, our observations indicate that mCNTs may undergo interorganelle movement through mechanisms such as lipid trafficking (*31*–*33*) and “kiss-and-run” interactions (*34*, *35*).

Having discovered mCNT distribution at mitochondria, we investigated whether mCNT + magnetic treatment affects mitochondria membrane potential, the perturbation of which can lead to cell death. We detected the dissipation of mitochondria membrane potential of GBM cells upon mCNT + magnetic treatment (Fig. 1I and fig. S8). When mitochondria integrity is compromised, mitochondrial cytochrome C is released into the cytosol to initiate caspases-dependent apoptosis (*36*, *37*). Consistent with altered mitochondria membrane potential, we observed increased cytosolic cytochrome C in GBM cells after mCNT + magnetic treatment (Fig. 1J and fig. S8). While mCNT treatment likely affects multiple intracellular organelles, these results provided evidence that mitochondrial perturbation underlies mCNT treatment–induced GBM cell death.

### mCNT distribution in GBM

**Fig. 2.**
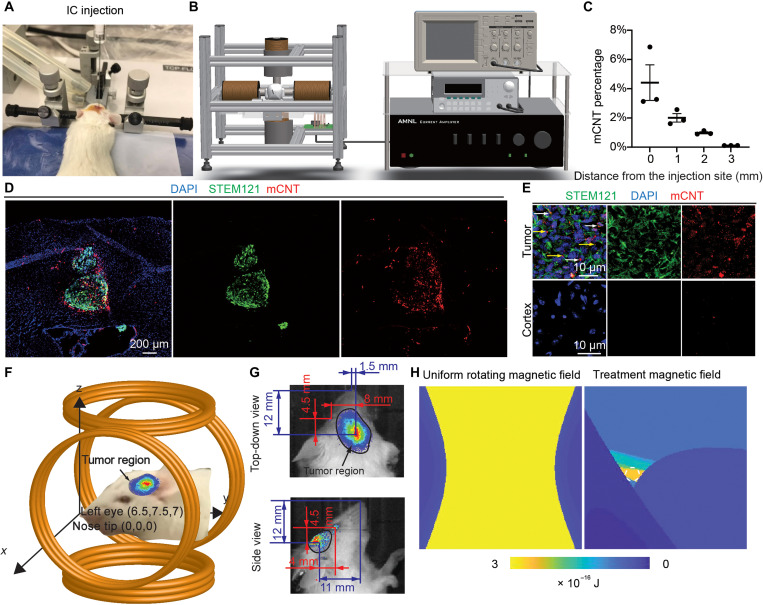
Magnetic treatment of GBM-bearing mice. (**A**) Intracranial (IC) injection of GBM stem cells into mouse brain. The same IC injection procedure is used to deliver mCNTs. (**B**) Magnetic field generation system for treating GBM-bearing mice. (**C**) mCNT percentage over the distance away from the injection site, measured based on histology images taken 24 hours after mCNT injection. mCNT percentage is quantified as the percentage of mCNTs per image (i.e., mCNT pixel numbers over total pixel numbers per image). *n* = 3 independently collected images. Error bar: SD. (**D** and **E**) Representative immunohistochemistry images of the tumor harvested 24 hours after mCNT injection. In (E), white arrows indicate mCNTs located in cytoplasm, and yellow arrows indicate mCNTs located in extracellular space. (**F**) Tumor location is translated into Cartesian coordinates using the nose and eyes’ locations. (**G**) Bioluminescence images (BLIs) of brain tumor size and its location relative to the nose tip and eyes of the mouse in top-down and side views. (**H**) Energy dissipation map in the workspace. White dashed circle indicates the tumor region.

Next, we investigated the distribution of mCNTs delivered intratumorally into GBM-bearing mice. Xenograft GBM tumors were generated by orthotopically implanting patient-derived GBM cells (G411) into the brains of immunocompromised mice (Fig. 2A). Approximately 7500 tumor cells were injected into the brain of each mouse. Tumor growth was monitored by bioluminescent imaging. Once substantial tumor burdens were detected, we introduced mCNTs into the tumors (Fig. 2B). mCNT solution of 1 mg/ml in concentration was intratumorally injected at the coordinates used for xenograft. We delivered a total mCNT amount of 5 μg for the ~7500 tumor cells orthotopically injected into each mouse, a level equivalent to 25 μg of mCNTs per ~37,500 cells (1 ml of mCNT solution with a concentration of 0.025 mg/ml) used in our in vitro experiments. To study mCNT distribution in GBM tumors, we harvested mouse brains 24 hours after mCNT injection and performed immunostaining to visualize mCNTs and tumor cells marked by human-specific antigen STEM121 (Fig. 2, C to E). We found that 53.83 ± 5.79% of mCNTs were located in the extracellular space of tumor tissues, while the remaining mCNTs were located either on the cell membrane or within the cytoplasm of tumor cells (*n* = 3 tumors from three mice; Fig. 2E).

### Magnetic field control for in vivo GBM treatment

Having determined intratumoral distribution of mCNTs, we developed a magnetic field generation system to mobilize mCNTs in a spatiotemporally controlled manner. The system includes a programmable function generator to control the current supplied to magnetic coils, a custom-designed current amplifier to increase current output, four magnetic coils with magnetic cores to generate a magnetic field, and an oscilloscope to monitor the generated waveform during treatment (Fig. 2B).

In the workspace of the magnetic system (Fig. 2, F and G), GBM region was determined by coordinate transformation from stereotaxic coordinates to Cartesian coordinates through the top-down view and side view of bioluminescence images (section S3 in the Supplementary Materials). The treatment relies on delivering rotational energy through mobilizing mCNTs by the magnetic field to generate mechanical torque (fig. S1, D to F). To reduce mCNT impact on nontumoral brain tissues, instead of generating a uniform rotating magnetic field, we designed a tumor region–focused magnetic field by delivering sufficient rotational energy to the tumor while minimizing the energy delivered to tumor-surrounding regions. In the first quarter of a control cycle (0 to ¼ T), one pair of adjacent coils functions as dominant coils, and the other pair of coils acts as auxiliary coils to attenuate the magnetic field outside the target region. In the second quarter of the same cycle (¼ to ½ T), the dominant and auxiliary coils are shifted clockwise by one coil. In this quarter, the magnetic field distribution changes, and the tumor-surrounding tissues treated in the previous quarter are now located in a region with low magnetic field strength. Dominant coils shift four times to complete a full control cycle. Throughout one cycle, the magnetic field strength in the tumor region is constantly large, while the magnetic field outside the tumor region is subjected to periodic (a quarter of the cycle) high and low magnetic field strength. In the tumor region, magnetic field strength was kept at 20 ± 2 mT throughout the cycle (fig. S9). The electric current waveforms supplied to four coils are shown in fig. S9A. Using finite element simulation (section S4 in the Supplementary Materials) to calculate the rotational energy delivered to the workspace, we determined that, for a uniform rotating magnetic field (20 ± 2 mT in field strength and 20 Hz in rotating frequency), 2.55 × 10^−16^ J per hour rotational energy is delivered by the mobilization of mCNTs to both tumor and tumor-surrounding regions. In contrast, our tumor-focused magnetic field delivers 2.50 × 10^−16^ and 0.16 × 10^−16^ J per hour rotational energy to tumor and tumor-surrounding regions, respectively (Fig. 2H), thereby allowing a ~15.6-fold difference in energy input to mobilize the mCNTs.

### Effect of magnetic treatment on GBM growth and survival of GBM-bearing mice

**Fig. 3.**
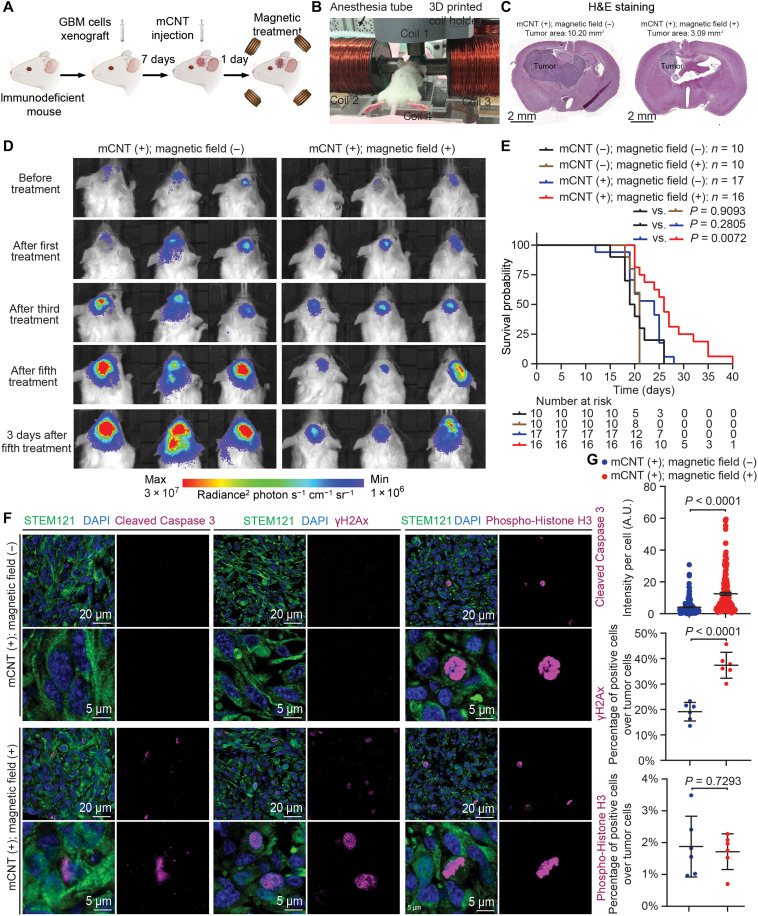
Magnetic treatment extends the survival of GBM-bearing mice. (**A**) Treatment protocol. GBM stem cells G411 are implanted into the mouse brain through IC injection. After 7 days of tumor growth, mCNTs are injected into the tumor location. Magnetic field is applied to the mouse the day after. (**B**) A mouse undergoing magnetic treatment. 3D, three-dimensional. (**C**) Representative hematoxylin and eosin (H&E) images. The stained sections shown in the figure represent the largest tumor area in each tumor. (**D**) Tumor size change of control and treatment groups monitored by BLI. (**E**) Survival comparison among control and treatment groups. *P* value is calculated by Kaplan-Meier analysis. (**F**) Immunohistochemistry of tumors harvested after the fifth treatment. Apoptosis marker cleaved Caspase 3, DNA damage marker γH2Ax, and mitosis marker phospho-Histone H3 are shown. (**G**) Quantification of immunohistochemistry results. *n* = 200 cells for cleaved Caspase 3, *n* = 6 independently stained slides for γH2Ax, and *n* = 6 independently stained slides for phospho-Histone H3. Error bar: SD. A.U., arbitrary units.

Next, we set to determine the therapeutic efficacy of magnetically mobilizing mCNTs to treat GBM in mice. For all GBM-bearing mice, we began magnetic field treatment 24 hours after mCNT injection. Each mouse was treated for 30 min while being maintained under anesthesia with body temperature and breathing pattern monitored. The 30-min treatment session was performed once every 2 days for a total of five times. We monitored tumor burdens by bioluminescence imaging (BLI). The mouse endpoint was defined as body weight loss reaching 20% (Fig. 3, A and B). The control group (mCNT^+^, magnetic field^−^) received anesthesia but no magnetic field treatment. The magnetic treatment group (mCNT^+^, magnetic field^+^) received tumor-focused magnetic field treatment. Compared to the control, the magnetic treatment group showed a notable decrease in tumor size (Fig. 3, C and D). Figure S10A summarizes the tumor area calculated from hematoxylin and eosin (H&E) staining after the fifth treatment, 8.22 ± 1.78 mm^2^ in the control group versus 3.71 ± 0.67 mm^2^ in the treatment group (*P* = 0.0353, *n* = 3 independently treated mice, error bar: SD). Figure 3C shows representative H&E-stained histological images. Furthermore, we determined tumor burden using noninvasive BLI before and immediately after magnetic treatment. BLI signal fold change (i.e., the signal immediately after magnetic treatment divided by the signal before treatment) was 0.52 ± 0.17 in the treatment group, compared to 1.06 ± 0.25 in the control group that only received anesthesia for 30 min (*n* = 3 independently treated mice, *P* = 0.0419; fig. S10, B and C). Consistent with tumor burden reduction, mCNT + magnetic field treatment significantly extended the survival of GBM-bearing mice (median survival: 22.2 ± 4.0 versus 26.8 ± 6.0 days, *P* = 0.0072; Fig. 3F). To determine the effect of mCNT injection alone or magnetic field application alone, two other control groups, including PBS injection with or without magnetic field treatment were characterized. Neither mCNT injection alone (*P* = 0.2805) nor magnetic field treatment alone altered mouse survival (*P* = 0.9093).

To investigate the treatment effects at the cellular level, we harvested the mouse brains of each group at the median survival time point of the control group mice and performed immunohistochemical analyses. STEM121 was used to distinguish xenografted human tumor cells from mouse cells. Compared with the control group that only received mCNT injection, tumor cells in the treatment group (mCNT^+^, magnetic field^+^) displayed significantly increased apoptosis (Fig. 3, F and G). Tumor cells in the treatment group also exhibited increased DNA damage (Fig. 3, F and G), demonstrating mCNT-induced mechanical perturbation of GBM cell nuclei (*38*). No difference in tumor cell mitosis was observed between the control and treatment groups (Fig. 3, F and G).

### mCNT functionalization using anti-CD44 antibody 

**Fig. 4.**
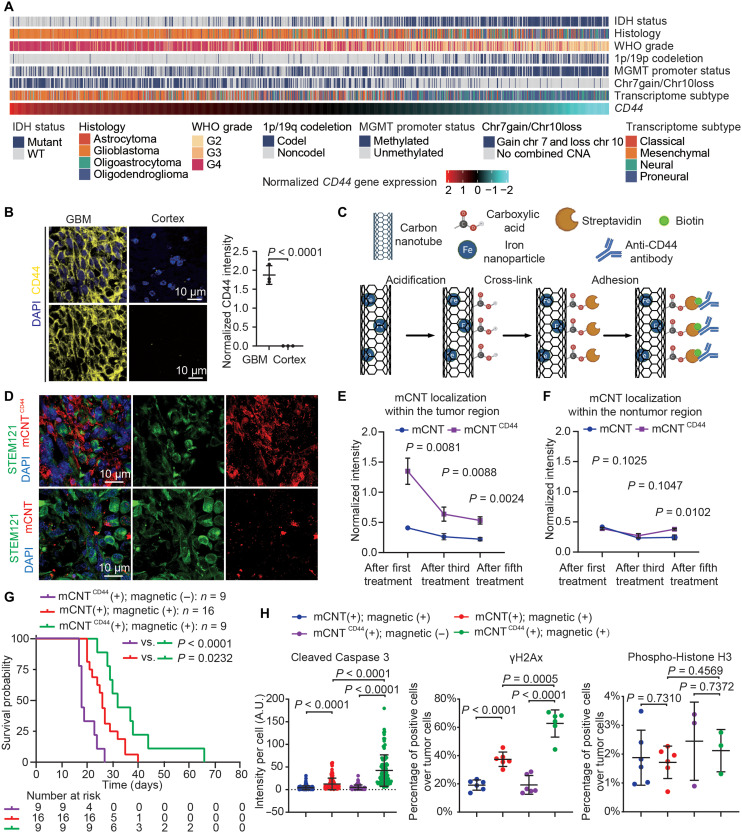
Anti-CD44 antibody functionalization increases treatment efficacy. (**A**) Heatmap of *CD44* expression with IDH status, histology, World Health Organization (WHO) grade, 1p/19p codeletion, O^6^-methylguanine methyltransferase (MGMT) promoter status, TERT promoter status, Chr7 gain/Chr10 loss, transcriptomic GBM subtype, DNA methylation cluster, as well as age and gender of patients with glioma. WT, wild type. (**B**) CD44 immunohistochemistry of G411 xenograft tumors surrounding cortex tissue. (**C**) Protocol for functionalizing mCNTs with anti-CD44 antibody. (**D**) Immunohistochemistry of tumors harvested after the third treatment with mCNTs or with mCNTs^CD44^. (**E** and **F**) Distribution of mCNTs (E) and mCNTs^CD44^ (F) is quantified after the first, third, and fifth treatment. *n* = 3 mice. Error bar: SD. (**G**) Survival comparison among the group that only receives mCNT^CD44^, the group that receives mCNTs and magnetic field treatment, and the group that receives mCNT^CD44^ and magnetic field treatment. *P* value is calculated using Kaplan-Meier analysis. (**H**) Quantification of immunohistochemistry results of tumor harvested after the fifth treatment. *n* = 200 cells for cleaved Caspase 3, *n* = 6 independently stained slides for γH2Ax, and *n* = 6 independently stained slides for phospho-Histone H3. Error bar: SD.

Next, we sought to enhance the ability of mCNTs to recognize GBM cells to increase their therapeutic utility. To this end, we interrogated The Cancer Genome Atlas low-grade glioma-GBM (LGG-GBM) dataset (*n* = 607) and found that high *CD44* expression is enriched in GBM [World Health Organization (WHO) grade 4] compared with LGGs (WHO grades 2 and 3). While *CD44* expression is detected across transcriptomic subtypes of gliomas, the highest *CD44* expression is present in mesenchymal GBM which portends the worst prognosis (*39*). Histologically, high *CD44* expression is detected in GBM, and less frequently in astrocytoma, oligodendroglioma, or oligoastrocytoma. Molecularly, high *CD44* expression is associated with gliomas with wild-type (WT) IDH and chromosome 1p/19q noncodeletion, which are biomarkers for aggressive gliomas. In accordance, high *CD44* expression is associated with shorter survival of patients with glioma (fig. S11). Because TMZ is the mainstay chemotherapy for treating patients with GBM, a major reason for TMZ resistance is the activity of O^6^-methylguanine methyltransferase (MGMT), which repairs O^6^-methylation DNA damage induced by TMZ. Methylation of MGMT promoter suppresses MGMT expression, thereby increasing GBM cell sensitivity to TMZ. High *CD44* expression correlates with unmethylated MGMT promoter, suggesting that CD44^+^ glioma cells confer TMZ resistance. Collectively, these data show that human gliomas with high *CD44* expression display histological and molecular features of aggressive and TMZ-resistant tumors (Fig. 4A). These data are consistent with previous reports showing that *CD44* expression is enriched in malignant gliomas (*39*–*41*). Using CD44 antibody that recognizes both human and mouse proteins, we detected prominent CD44 expression in xenografted GBM cells, while low CD44 expression was found in nontumoral cells (Fig. 4B). Hence, we chose CD44 for mCNT functionalization.

In conventional CNT functionalization, the first step is to treat CNTs with a strong acid, such as hydrochloric acid, to produce openings of carbon-carbon bonds (*25*). However, strong acids cannot be used here to avoid dissolving iron particles inside mCNTs. Therefore, we used citric acid to produce openings in carbon-carbon bonds and functionalized mCNT surface with a carboxyl group (Fig. 4C) (*42*). Streptavidin-amine was integrated into the carboxyl group on mCNT surface, resulting in a streptavidin-functioned surface. Relying on streptavidin-biotin binding, surface protein antibody was integrated onto mCNT surface. We used Alexa Fluor 555–streptavidin to visualize the cross-link of streptavidin onto mCNTs. Robust Alexa Fluor 555–streptavidin signal was observed, demonstrating successful functionalization of streptavidin to mCNTs (fig. S12A). Last, we integrated biotinylated mouse anti-CD44 antibody onto the streptavidin-mCNTs. Immunostaining using anti-mouse Alexa Fluor 488 secondary antibody confirmed successful functionalization of anti-CD44 antibody onto mCNTs.

Next, we incubated GBM cells (G411) with the same amount of CD44-functionalized mCNTs (mCNT^CD44^) and unfunctionalized mCNTs for 24 hours. Notably, mCNT^CD44^ demonstrated a higher internalization rate compared to mCNTs (fig. S12B) and induced a higher cell death rate with magnetic field application (fig. S12C). We then investigated whether CD44 functionalization results in increased mCNT enrichment and retention in the tumors of GBM-bearing mice. We injected the same amount of mCNT^CD44^ and unfunctionalized mCNTs into xenografted tumors 7 days after tumor cell implantation. We treated mice using the same in vivo treatment protocol as described in Figs. 2 and 3. At the time points of the first, third, and fifth treatment, we harvested mouse brains and compared the distributions of mCNTs (Fig. 4, D to F). The mCNTs were observed to form aggregates in vitro and in vivo (Figs. 1G and 4D). Compared to unfunctionalized mCNT, significantly higher amounts of mCNT^CD44^ were detected in the tumor region at all three time points (Fig. 4E); at the end of the fifth treatment, the amount of mCNT^CD44^ retained in the tumor was 2.42 ± 0.37–fold higher than unfunctionalized mCNTs (*n* = 3 mice). In the nontumor region, the normalized intensity of both mCNT and mCNT^CD44^ was lower than 0.5 at all three time points (Fig. 4F). We further investigated the size distribution of mCNT and mCNT^CD44^ within tumor tissues in vivo. At the end of the first, third, and fifth treatment, as summarized in fig. S13, the sizes of mCNT aggregates were 0.282 ± 0.009, 0.240 ± 0.012, and 0.198 ± 0.011 μm, respectively (*n* = 300 aggregates at each time point, error bar: SE); and the sizes of mCNT^CD44^ aggregates were 0.325 ± 0.013, 0.256 ± 0.011, and 0.166 ± 0.010 μm, respectively (*n* = 300 aggregates at each time point, error bar: SE). Over time, nanomaterials may be engulfed by microglia (*43*, *44*), transported by cerebellum fluid *(**45*, *46*), and circulated toward the kidney and intestine for excretion through the biliary and renal pathways (*47*, *48*).

Having established the ability of surface functionalization to enrich mCNTs, we then determined the therapeutic efficacy of mCNT^CD44^ in treating GBM. Mice in the mCNT^CD44^ treatment group displayed significantly longer survival than mice that received mCNT^CD44^ without magnetic field treatment (*P* < 0.0001; Fig. 4G) or mice in the unfunctionalized mCNT treatment group (*P* = 0.0232; Fig. 4G). To investigate the treatment effects at the cellular level, mouse brains were harvested and processed for immunohistochemical analyses. We detected significantly increased apoptosis and DNA damage in tumor cells of mCNT^CD44^ treatment group compared to the unfunctionalized mCNT treatment group. No difference in tumor cell mitosis was observed (Fig. 4H). These results demonstrated that the functionalization of mCNTs with anti-CD44 antibodies enhanced their therapeutic efficacy in treating GBM.

Anti-CD44 functionalization of mCNTs was designed to target mesenchymal GBM which portends the worst prognosis among all GBM subtypes. Through immunocytochemistry staining on GBM stem cells derived from two different subtypes (mesenchymal: G411, G532, and G508 and perineural: G523 and G549), we observed significant higher CD44 expression in GBM cells derived from the mesenchymal subtype (fig. S14, A and B). Consistently, mCNT^CD44^ + magnetic field treatment demonstrated superior efficacy in killing G411, G532, and G508 (*P* = 0.0216, 0.0464, and 0.0239, respectively) but not G523 and G549 (*P* = 0.8900 and 0.1187, respectively) (fig. S14C). We note that anti-CD44 functionalization reported here serves as proof of principle for enhancing the treatment efficacy of our mCNT approach. Personalized functionalization protocol can be applied to incorporate other tumor cell surface antigens to benefit patients with heterogeneous GBM tumors.

### Efficiency of mCNT and mCNT^CD44^ treatment against TMZ-resistant GBM

TMZ, a DNA alkylating agent that causes single- and double-stranded DNA breaks (*49*), is a cornerstone therapy for patients with GBM worldwide. Because upfront or treatment-induced TMZ resistance underlies therapy failure, disease relapse, and patient mortality, we investigated whether mCNT^CD44^ treatment is effective against TMZ-resistant GBM.

To model primary TMZ resistance, we generated GBM cell lines with CRISPR-induced knockout of *MSH6*, which encodes a DNA mismatch repair gene frequently mutated in TMZ-resistant glioma (*50*–*52*). To model treatment-induced TMZ resistance, we cultured GBM cells in increasing dosages of TMZ, followed by selecting and establishing resistant cell lines (*3*). Two resistant cell lines of each TMZ resistance paradigm were established.

**Fig. 5.**
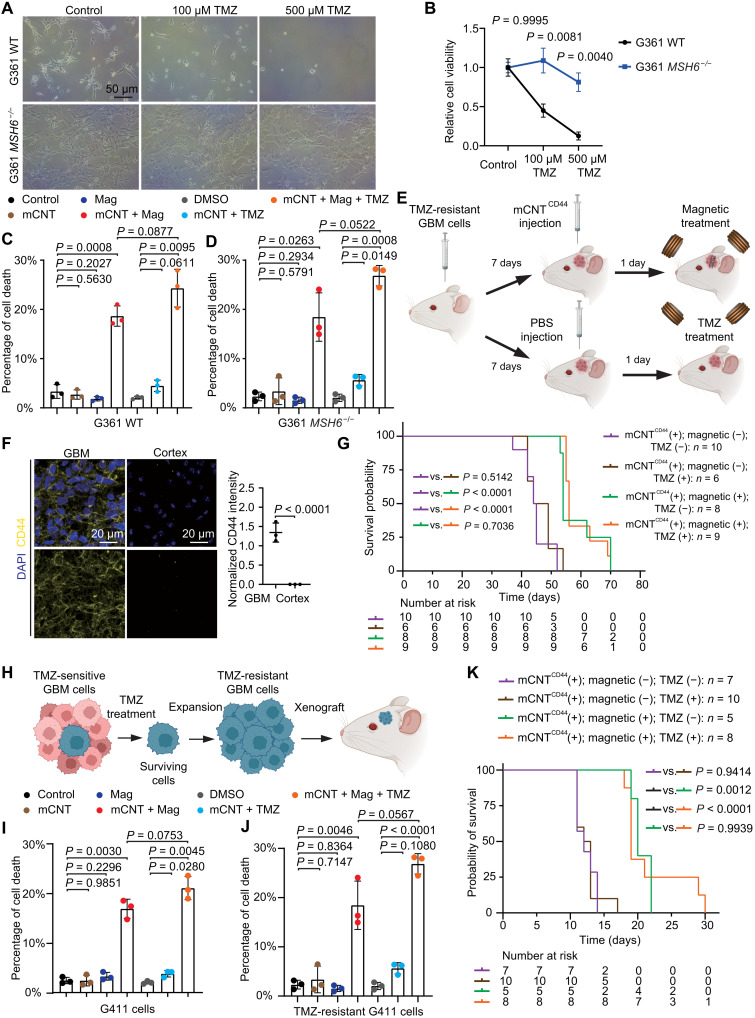
mCNT treatment is effective against chemotherapy-resistant GBM. (**A**) G361 parental and G361 *MSH6*^−/−^ cells were treated with dimethyl sulfoxide (DMSO; control) as well as 100 and 500 μM temozolomide (TMZ). (**B**) Relative cell viability after treating cells with DMSO (control) as well as 100 and 500 μM TMZ for 5 days. *n* = 3 independent experiments. Error bar: SD. (**C** and **D**) Cell death rate for cells treated with mCNTs, magnetic field treatment, TMZ, and their combinations for G361 parental (C) and G361 *MSH6*^−/−^ cells (D). *n* = 3 independent experiments. Error bar: SD. Mag, Magnetic. (**E**) Treatment protocol for applying mCNTs^CD44^ and magnetic treatment and TMZ chemotherapy. (**F**) CD44 staining of tumors developed from G361 *MSH6*^−/−^ cells. (**G**) Survival comparison of G361 *MSH6*^−/−^ tumor-bearing mice with mCNTs^CD44^ magnetic field treatment, TMZ treatment, and their combinations. *P* value is calculated using Kaplan-Meier analysis. (**H**) TMZ treatment for generating chemoresistant cells. (**I** and **J**) Cell death rate for cells treated with mCNTs, magnetic field treatment, TMZ, and their combinations for G411 parental (I) and TMZ-resistant G411 cells (J). *n* = 3 independent experiments. Error bar: SD. (K) Survival comparison of TMZ-resistant G411 tumor-bearing mice with mCNTs^CD44^ magnetic field treatment, TMZ treatment, and their combinations. *P* value is calculated using Kaplan-Meier analysis.

We first studied two GBM stem cell lines (G361 and G440) and their *MSH6* CRISPR knockout counterparts. As expected, parental, but not *MSH6^−/−^*, G361 cells displayed reduced cell viability upon TMZ treatment (Fig. 5, A and B). Notably, mCNTs with magnetic field treatment significantly increased cell death rate, with or without TMZ treatment, in both parental and *MSH6^−/−^* G361 cells (Fig. 5, C and D). Similarly, mCNT, but not TMZ, treatment displayed robust efficacy in inducing cell death in both parental and *MSH6^−/−^* G440 cells (fig. S15). We orthotopically implanted G361 *MSH6^−/−^* cells to generate TMZ-resistant GBM (Fig. 5E). Similar to G411 xenograft tumors (Fig. 4B), pronounced CD44 expression is present in G361 *MSH6^−/−^* tumor cells but not nontumoral cells (Fig. 5F). Seven days after tumor cell implantation, mCNT^CD44^ were injected into the tumor, followed by TMZ, magnetic, or combination treatment (Fig. 5E). The control group received dimethyl sulfoxide (DMSO; as opposed to TMZ) and anesthesia (as opposed to magnetic field) treatment. No survival difference was observed between the control group and the group that received TMZ treatment alone, demonstrating that these tumors are TMZ-resistant (Fig. 5G). Mice with mCNT^CD44^ + magnetic field treatment, with or without TMZ treatment, displayed significantly extended survival compared to the control. No difference was observed between the groups (G361 and G440) with mCNT^CD44^ + magnetic field treatment with or without TMZ chemotherapy.

We next studied the therapeutic efficacy of mCNT^CD44^ treatment against GBM with treatment-induced TMZ resistance (Fig. 5H). TMZ-resistant G411 and G532 GBM cells, which were established by culturing the cells with escalating dosages of TMZ and selecting survival clones, demonstrated robust TMZ resistance compared to their parental cell lines (fig. S15). First, we determined that mCNT + magnetic treatment induced robust cell death in both parental and TMZ-resistant cell lines in vitro (Fig. 5, I and J, and fig. S15). Next, we orthotopically implanted TMZ-resistant G411 cells to generate tumors in mice. Seven days after tumor cell implantation, we intratumorally injected mCNT^CD44^, followed by TMZ treatment, magnetic field treatment, or both. mCNT^CD44^ + magnetic field treatment, but not TMZ, significantly extended mouse survival (Fig. 5K). Last, we investigated the potential side effects of mCNT treatment and performed a pathohistological study of major internal organs of mCNT-treated mice. No overt histological changes were detected in the heart, liver, kidney, lung, or spleen (fig. S16). Together, these results demonstrated the utility of using mCNT^CD44^ + magnetic field treatment to mitigate TMZ-resistant GBM.

mCNTs were observed to be localized within the extracellular space, on cell membrane, and within the cell. As illustrated in fig. S17, we propose that mechanical torque from magnetic actuation can be transmitted from extracellular space to inside of the cell, generated directly on cell membrane, and generated within the cell to mechanically damage intracellular organelles (e.g., lysosomes, mitochondria, and nucleus; Fig. 1, I and J), increase DNA damage (Fig. 3, F and G), and induce cell apoptosis (Fig. 3, F and G). TMZ preferentially methylates DNA at N^7^ position of guanine, O^3^ position of adenine, and O^6^ position of guanine (*53*, *54*). Alkylation of the O^6^ site on guanine leads to the formation of O^6^-methylguannine adducts and results in the insertion of thymine residues instead of cytosine (*53*, *54*). These unrepairable mutations induce the formation of single- and double-stranded DNA breaks, resulting in cell cycle arrest at G_2_/M and apoptosis (*55*). In contrast, our mCNT + magnetic field treatment approach is principally unrelated to a specific biochemical process, such as inducing DNA damage, to induce therapeutic effects. Hence, we demonstrate that our mechanical nanosurgery approach has robust efficacy in treating TMZ-resistant GBM.

## DISCUSSION

Despite aggressive standard treatment that combines surgical resection, radiation, and TMZ-based chemotherapy, the median survival of patients with GBM has remained at ~15 months for decades (*2*). Targeting selected proteins or biochemical pathways either promotes the expansion of preexisting therapy-resistant tumor cell clones or drives tumor evolution toward acquiring mutations that confer de novo resistance, leading to GBM recurrence and patient death (*56*, *57*). Recognizing that GBM has such an extreme level of resilience to biochemically targeted therapies, we developed a mechanical nanosurgery approach by inducing GBM cell death through magnetically mobilizing mCNTs delivered to the tumors. We show that the functionalization of mCNTs using an antibody to recognize a GBM cell surface antigen further elevated the treatment efficacy. We also demonstrate that our mechanical nanosurgery approach has robust efficacy in treating TMZ-resistant GBM.

Treating brain tumors with physical approaches is an emerging area (*58*–*60*). For example, U.S. FDA-approved Optune therapy uses alternating electric fields, named tumor-treating fields (TTFs) to treat recurrent GBM as a monotherapy or in combination with chemotherapy for newly diagnosed GBM. TTF mechanisms involve the disruption of mitotic spindles and cell division by electric field–induced dipole alignment and dielectrophoresis (*61*, *62*). In a case report, Baskin *et al*. (*60*) described a patient with recurrent GBM treated with an oscillating magnetic field for 5 weeks, which was associated with a 31% reduction of contrast-enhanced tumor volume and a reduction in abnormal T2-weighted fluid-attenuated inversion recovery volume. Moreover, 5 weeks of such magnetic field treatment was well tolerated in the patient. Because this report described a single patient, the efficacy and safety of using magnetic field to treat GBM requires further investigation in patients. Of note, we did not detect therapeutic effects after applying a rotating magnetic field without the introduction of mCNTs either in vitro or in vivo.

In this study, we demonstrated the utility of using mCNT + magnetic field to treat GBM in mouse cortex. We envision that our approach may be adapted to debulk tumors in other brain regions or multifocal tumors in the central nervous system. Our approach may also be applied to inoperable brain tumors situated at vital regions (i.e., brainstem) that control life-sustaining functions such as breathing and cardiac functions (*63*). Because multifocal or inoperable tumors cannot be readily removed by conventional surgery, our study provides a strong impetus for future investigations to determine the utility of mCNT-based mechanical nanosurgery in treating these challenging tumor types. Last, because we provide proof of principle that mCNT functionalization using a GBM cell–recognizing antibody enhances mCNT retention in the tumors and its therapeutic efficacy, versatile functionalization strategies can be devised. Increasing the tumor-homing ability of mCNTs by various tumor recognition molecules that target heterogeneous tumor cell populations or conjugating mCNTs with chemotherapy, targeted therapy, or immunotherapy represent promising avenues for future investigations.

## METHODS

### Patient-derived cell lines

Patient-derived cell lines (G361, G411, G440, G508, G523, G532, and G549) were established at The Hospital for Sick Children, and all samples were obtained following informed consent from patients. All experimental procedures were performed in accordance with the Research Ethics Boards at The Hospital for Sick Children (Toronto, Canada) and the University of Toronto and The Centre for Phenogenomics (TCP) Animal Care Committee. G361 and G440 MSH6 knockout cell lines were generated as previously described (*64*). G: GBM stem cell lines.

### Cell culture conditions

GXXX samples were grown adherently in serum-free medium as described in (*65*). Briefly, cells were grown on Primaria culture plates (Corning) coated with poly-l-ornithine (Sigma-Aldrich) and laminin (Sigma-Aldrich) and maintained in NeuroCult NS-A basal medium (human) (STEMCELL Technologies) containing 2 mM l-glutamine (Wisent), bovine serum albumin (75 μg/ml; Life Technologies), in-house hormone mix equivalent to N_2_ (homemade), B27 supplement (Life Technologies), recombinant human epidermal growth factor (10 ng/ml; Sigma-Aldrich), basic fibroblast growth factor (10 ng/ml; STEMCELL Technologies), and heparin (2 μg/ml; Sigma-Aldrich). Cells were passaged using enzymatic dissociation with Accutase (STEMCELL Technologies).

### mCNT preparation

The CNTs were filled with iron through the excessive-catalyst injection chemical vapor deposition (CVD) method (*66*). In brief, a solution of ferrocene dissolved in xylene was intermittently fed into a horizontal quartz tube in a tubular electrical furnace. Once CVD temperature reached 800°C, the solution was injected. A mixture of hydrogen and nitrogen was used as carrier gas. The ferrocene concentration in xylene was 0.2 g/ml, which is an order of magnitude higher than the usual 0.02 g/ml used in CNT synthesis. The growth time was 30 min. The size of mCNTs can be regulated by modulating the growth time during mCNT fabrication, and the concentration of iron can be increased by elevating ferrocene concentration in xylene. After injection, the furnace was cooled to room temperature, and the collected mCNTs were treated with sonication in PBS for generating smaller lengths and then imaged by TEM and SEM. The results showed that the diameter of iron particles is 16.25 ± 3.24 nm and the roundness of iron particles is 0.75 ± 0.09. The overall magnetic coercivity Hc of mCNT was approximately 700 Oe. The pore size of mCNTs was smaller than 5 nm. mCNT outer and inner diameters are 70.77 ± 7.24 and 25.19 ± 6.31 nm, respectively (*n* = 30 mCNTs analyzed under TEM, error bar: SD).

mCNT PBS or ethanol suspension was prepared by a standard sonication method. Specifically, 10 mg of mCNT powder (NG01SC0703-5-CNT, Nanografi Nano Technology Co. Ltd.) in 10 ml of PBS or ethanol was sonicated in an ice bath using a Sonic Dismembrators (FB432B2, Fisher Scientific) for 30 min (15 W and 20 kHz). mCNT PBS suspension was used immediately, while mCNT ethanol suspension was stored for further functionalization.

### mCNT functionalization

mCNTs were coated with biotin anti-CD44 antibodies (Abcam, ab157105) or fluorescent Alexa Fluor 555 streptavidin (Thermo Fisher Scientific, S32355) through a three-step method. mCNTs functionalized with carboxyl (─COOH) groups were first obtained from citric acid–assisted O_2_ plasma treatment. Specifically, 10 ml of mCNT ethanol suspension was dried under vacuum for 2 hours. Dry mCNTs were collected and immersed in 5 ml of citric acid aqueous solution (0.1 M) for 48 hours at room temperature. The suspension was then treated by O_2_ plasma for 10 min (5 standard cubic centimeters per minute of O_2_, 400 Pa, and 200 WRF power). After plasma treatment, the suspension was washed to pH 7 with distilled water and dried. The carboxyl mCNT was then coupled with 1-ethyl-3-(3-dimethylaminopropyl)carbodiimide hydrochloride1 (20 mM) (MilliporeSigma, E7750) and *N*-hydroxysuccinimide sodium salt (40 mM) (MilliporeSigma, 56485) in an MES buffer solution (pH 5.5) and sonicated in a water bath sonicator for 30 min. Rinsed with PBS three times, the amine-reactive mCNT was lastly transferred to biotin anti-CD44 antibodies PBS solution (10 μg/ml) or streptavidin PBS solution (10 μg/ml) and stored for 3 hours. After a final wash with cold PBS, mCNTs coated with antibodies or dyes were used for treatment or imaging.

### Quantification of cell death rate

Trypan blue exclusion assay was used to evaluate cell viability. After treatment, cells were seeded in a petri dish at a density of 2 × 10^5^ cells per dish and maintained in cultures for 24 hours. Cells incubated at each time interval were washed twice with PBS before treating with Accutase, following which cell suspensions were mixed with an equal volume of 0.4% trypan blue. Ten microliters of stained cells were placed in Countess Cell Counting Chamber Slides, and the number of viable cells was counted. Three independent experiments were performed. Trypan blue staining detects all forms of cell death (*67*). The caspase staining was used to determine cell death through apoptosis.

### Animal studies

All mouse procedures were performed in compliance with Animals for Research Act of Ontario and the Guidelines of the Canadian Council on Animal Care. Mouse experimental procedures were approved by The Centre for Phenogenomics Animal Care Committee. Mice were housed at The Centre for Phenogenomics, Toronto, Ontario.

### Xenograft

Eight-week-old female *NOD scid gamma/J#5557* immunodeficient mice were used for xenograft experiments. Mice were housed under aseptic conditions, including filtered air and sterilized food, water, bedding, and cages. Mice were randomly assigned to experimental groups. Mice were anesthetized using gaseous isoflurane and immobilized in a stereotaxic head frame. The skull of the mouse was exposed, and a small opening was made using a sterile dental drill (Precision Guide) at 2 mm lateral and 3 mm posterior to lambda. At this location, ~7500 G411 cells in 3 μl of culture media were slowly injected (over 3 min) 2 mm deep beneath the surface of the skull using a 26-gauge Hamilton syringe. All procedures were performed under sterile conditions. Time-matched tumors were collected and processed for immunohistochemistry.

### In vivo bioluminescence monitoring

Tumor cells were engineered to express firefly luciferase. Mice were anesthetized using gaseous isoflurane and imaged 10 min after intraperitoneal injection of luciferin. The bioluminescence signal was quantified within a region of interest over the head defined by the Living Image Software.

### TMZ treatment

After tumor engraftment was confirmed by BLI, TMZ (25 mg/kg) was administered via gavage (VWR no. 7092 metal gavage needle, straight, 3.81 cm long, ball diameter of 2.25 mm) daily for 7 or 14 days (depending on the median survival of the xenograft tumor model) alone or combined with mCNT treatment.

### Transmission electron microscopy

Mice were anesthetized, and perfused with PBS followed by EM-grade fixative (4% paraformaldehyde and 2.5% glutaraldehyde in 0.1 M cacodylate buffer). Mouse brains were harvested and fixed by immersion in fixative overnight at 4°C. Tissues were rinsed in cacodylate buffer, postfixed in 1% osmium tetroxide in buffer, dehydrated in a graded ethanol series (50, 70, 90, and 100%), followed by propylene oxide, and embedded in Quetol-Spurr resin. Sections of 70 nm thickness were cut using a Leica EM UC7 ultramicrotome, stained with uranyl acetate and lead citrate, and imaged using an FEI Tecnai 20 TEM at 120 kV. The identification of the intracellular organelles was according to “cell and organelles Dr. Jastrow’s electron microscopic atlas” (*68*).

### Scanning electron microscopy

For SEM of cells, the cells were washed three times with buffer solution (0.1 M sodium cacodylate buffer with 0.2 M sucrose, pH 7.3) after cell culture media removal. Washing with buffer solution rather than PBS or cell culture media minimizes the salt left on the cell surface. Cells were then fixed by fixing solution (2% glutaraldehyde in 0.1 M sodium cacodylate buffer, pH 7.3) for 2 hours and washed with buffer solution for 20 min to remove the fixatives. The sample was dehydrated in a graded ethanol series (70, 90, 100, 100, and 100%) for 20 min in each solution. The sample was dried under a critical point dryer (BAL-TEC Critical Point Dryer 030) and sputter coated with a 6-nm layer of gold.

For SEM of tissues, mice were anesthetized and perfused with PBS, followed by EM-grade fixative (4% paraformaldehyde and 2.5% glutaraldehyde in 0.1 M cacodylate buffer). Mouse brains were harvested and fixed by immersion in the fixative overnight at 4°C. Tissues were rinsed in cacodylate buffer, postfixed in 1% osmium tetroxide in buffer, and dehydrated in a graded ethanol series (50, 70, 90, and 100%), followed by drying within a critical point dryer (BAL-TEC Critical Point Drying 030 Critical Point Dryer, Leica Biosystems). The sample was sputtered with 8 nm of gold (ICE sputter coater, Leica Microsystems) and imaged using a Philips/FEI XL30 scanning electron microscope.

### Immunocytochemistry

Immunocytochemistry was performed on cultured cells as previously described (*18*). Cells on coverslips were fixed for 15 min with 4% paraformaldehyde and permeabilized with 0.1% Triton X-100 in PBS (PBST). Cells were then blocked with 10% normal goat serum in PBST for 1 hour at room temperature and incubated with primary antibodies in blocking solution overnight at 4°C, followed by incubation with fluorophore-conjugated secondary antibodies (1:200 to 1:400) and 4′ and om temperature and inc (DAPI; 1 μg/ml; Sigma-Aldrich) for 1 hour at room temperature. For F-actin staining, cells were stained with 1:500 Alexa Fluor 488 Phalloidin (Invitrogen). Coverslips were mounted onto glass slides using ProLong Gold (Invitrogen). Primary antibodies include: anti-STEM121 (1:1000; Takara Bio Inc., Y40410), anti-cleaved Caspase-3 (1:1000; Cell Signaling Technology, 9661), anti-cathepsin B (1:1000; Cell Signaling Technology, 31718S), anti-γH2Ax (1:1000; Cell Signaling Technology, 2577S), anti–phospho-Histone H3 (1:1000; Cell Signaling Technology, 9706S), anti-LAMP1 (1:1000; Cell Signaling Technology, 9091P), anti-TOM20 (1:1000; Santa Cruz Biotechnology, sc-11415), anti-EEA1 (1:1000; Cell Signaling Technology, 3288p), and anti-cytochrome c (1:1000; Thermo Fisher Scientific, 11-6601-82). Images were acquired using a Leica SP8 Lightning Confocal DMI6000 microscope. Images were analyzed using Imaris software.

### Immunohistochemistry and image quantification

Immunohistochemistry and H&E staining were performed on cryo- or paraffin-embedded tissue sections. Primary antibodies were anti-STEM121 (1:100; Takara Bio Inc., Y40410), anti-cleaved Caspase-3 (1:200; Cell Signaling Technology, 9661), anti-cathepsin B (1:200; Cell Signaling Technology, 31718S), anti-γH2Ax (1:500; Cell Signaling Technology, 2577S), anti–phospho-Histone H3 (1:200; Cell Signaling Technology, 9706S), and anti-CD44 (1:200; Abcam, ab243894). Secondary antibodies conjugated to Alexa Fluor dyes (488, 555, and 647) at a dilution of 1:200 were used. Terminal deoxynucleotidyl transferase–mediated deoxyuridine triphosphate nick end labeling kit (Sigma-Aldrich, cat. no. S7110,) was used for apoptosis quantification. DAPI (Sigma-Aldrich, cat. no. D9564) was used for nuclear counterstain. Images were acquired using a Leica SP8 Lightning Confocal DMI6000 microscope. Images were analyzed using Imaris software.

### JC-1 assay for mitochondrial membrane potential

Membrane potentials of mitochondria were measured using JC-1 Mitochondrial Membrane Potential Assay Kit (Abcam) according to the manufacturer’s instructions. G411 cells (1 × 10^6^) were seeded in a 35-mm glass bottom dish (P35G-1.5-14-C, MatTek) and incubated for 24 hours to allow the cells to attach. mCNTs (0.05 mg/ml) were then added for 24 hours. After washing the cells with PBS, the cells were incubated with JC-1 solution for 10 min at 37°C. Cells of the treatment groups were treated with a magnetic field for 30 min. The cells were then imaged using a confocal microscope at an excitation of 535 ± 20 nm for aggregated JC-1 only or an excitation of 475 ± 20 nm for both aggregated and monomer forms of JC-1.

### Statistics and reproducibility

No statistical methods were used to predetermine the sample sizes. Statistical analyses were done afterward without interim data analysis. No data points were excluded. Two-tailed Student’s *t*-test was performed for comparison between two groups of samples. One-way analysis of variance (ANOVA) with Tukey’s multiple comparisons correction or Kruskal-Wallis test with Dunn’s multiple comparisons correction was used to analyze differences between multiple groups. Two-way ANOVA analyses with Geisser-Greenhouse correction were used to assess the significance of multiple data points. Kaplan-Meier estimator and GraphPad Prism software were used to generate survival curves. Differences between survival curves were calculated using a log-rank test. All data were collected and processed randomly. Each experiment was repeated at least three times and performed on different days. All measurements were taken from distinct samples. All data were expressed as means ± SD. *P* value less than 0.05 was considered statistically significant.

## Supplementary Materials

### This PDF file includes:

Supplementary Methods Sections S1 to S5 Figs S1 to S18 Legend for movie S1 References
