# Supplementary material for: Mechanical nanosurgery of chemoresistant glioblastoma using magnetically controlled carbon nanotubes
Source: Sci Adv. 2023 Mar 29;9(13):eade5321. doi: 10.1126/sciadv.ade5321 (PMC10058241; doi:10.1126/sciadv.ade5321)
Supplement: Supplementary file 1 — Supplementary Methods Sections S1 to S5 Figs S1 to S18 Legend for movie S1 References [file sciadv.ade5321_sm.pdf]

Science Advances
AAAS

Supplementary Materials for

Mechanical nanosurgery of chemoresistant glioblastoma using magnetically controlled carbon nanotubes

Xian Wang *et al.*

Corresponding author: Xi Huang, xi.huang@sickkids.ca; Yu Sun, yu.sun@utoronto.ca

Sci. Adv. **9**, eade5321 (2023)
DOI: 10.1126/sciadv.ade5321

The PDF file includes:

Supplementary Methods
Sections S1 to S5
Figs S1 to S18
Legend for movie S1
References

Other Supplementary Material for this manuscript includes the following:

Movie S1

Supplementary Methods

I. Comparison of two types of mCNTs

For mCNTs, magnetic materials such as iron particles can be either on the surface or inside the nanotubes. The toxicity effects and treatment effects of these two types of mCNTs were compared. Three concentrations of mCNTs (*n* = 3 independent repeated experiments for each of the three concentrations 0.01 mg/mL, 0.025 mg/mL, 0.05 mg/mL) were used in experiments and treated as a block condition in statistical analysis. The results showed a significant lower percentage of cell death ratio using mCNT2 (iron inside) compared with mCNT1 (iron on surface) (9.93 ± 3.21% vs. 22.14 ± 14.27%, error bar: standard deviation, $P = 0.0235$) (**Figure S1b**), demonstrating the lower toxicity to the cells from mCNT2.

The treatment effect under the same magnetic field treatment was quantified and compared between two types of mCNTs. The treatment effect was quantified as the subtraction between cell death ratio after magnetic treatment and cell death ratio without magnetic treatment (cell death solely caused by toxicity from mCNTs). Similar to the quantification of toxicity, three concentrations were used to treat the cells (*n* = 3 independent repeated experiments for each of the three concentrations 0.01 mg/mL, 0.025 mg/mL, 0.05 mg/mL) and used as blocking conditions in statistical analysis. The results showed no significant difference in percentage of cell death ratio caused by magnetic treatment using mCNT2 compared with mCNT1 (18.11 ± 5.75% vs. 19.98 ± 3.02%, error bar: standard deviation, $P = 0.4016$) (**Figure S1c**). mCNT2 with iron inside was chosen for all subsequent experiments.

II. Magnetic field parameters

When subjected to a magnetic field, mCNTs align with direction of the magnetic field (i.e., the direction of magnetic flux density B), and rotate with the rotating magnetic field. In magnetic field control, a mCNT align itself with direction of the magnetic field strength through torque, which depends on magnetic moment of the mCNT and magnitude of the magnetic field strength.

$$\mathrm{T} = \mathbf{m} \times \mathbf{B} = |\mathbf{m}||\mathbf{B}|\sin\theta \qquad (1)$$

where T is torque, **m** is magnetic moment on the mCNT which depends on magnetic property of the mCNT (e.g., concentration of iron) and mass, **B** is field flux density, and θ is angle between mCNT and the magnetic field. The rotation aligns **m** with **B**, minimizing θ until it reaches zero. When mCNTs are in the rotating magnetic field, the magnetic torque, determined by the magnitude of magnetic field flux density, rotates mCNT to follow rotation of the magnetic field. To estimate the rotating motion of mCNT, the mechanical work is

$$\mathrm{W}_{\mathrm{rotational}} = \frac{1}{2}\mathrm{I}\omega^2 \qquad (2)$$

where ω is angular velocity, I is moment of the inertia around the axis of rotation depending on the mass and geometry of mCNT (**Figure S1d**), $W_{\mathrm{rotational}}$ is mechanical work applied through the rotational motion. Based on the rotational energy equation, the angular frequency of the rotation of mCNT would determine the rotational energy delivered to cells over a 30-minute treatment duration.

The rotational motion of mCNTs applies mechanical stimulation to intracellular structures (e.g., intracellular membranes and cytoskeleton). Based on equations (1) and (2), the magnitude and rotating frequency of **B** affect the mechanical stimulation generated by mCNT. Magnetic treatment effect on GBM cells from magnetic field strength and rotation frequency was studied through a factorial design experiment with magnetic field strength of 10, 20, 40 mT, and frequency of 10, 20, and 30 Hz (other experimental conditions were kept the same: cells were cultured with mCNTs for 24 hours before treatment; the treatment was applied for 30 minutes for each magnetic field parameter; the experiments were conducted at room temperature). Under each condition, cells were randomly separated into control and treatment group after culturing with mCNTs for 24 hours. Cells in control group received no magnetic field treatment. Cell death ratio of control and treatment group was calculated through trypan blue staining, and treatment effect was quantified as cell death ratio in magnetic treatment group minus cell death in control group.

The results showed that the magnetic field with a magnitude of 20 mT field strength and a rotating frequency of 20 Hz had greatest treatment effect. The magnitude of 20 mT and 40 mT generates an insignificant cell death rate difference (14.35 ± 1.03% vs. 13.75 ± 2.63%, error bar: standard deviation, P = 0.7414, n = 3 independent experiments, **Figure S1e**). A smaller magnitude of 10 mT likely did not generate sufficient strong alignment torque to rotate mCNTs (cell death rate: 10.94 ± 0.86%, error bar: standard deviation, P = 0.0127, n = 3 independent experiments, **Figure S1e**). Compared with a lower frequency, the rotational energy delivered to cells was stronger using a higher magnetic rotating frequency, resulting in increased cell death rate after treatment (10 Hz vs. 20 Hz: 8.28 ± 2.53% vs. 12.64 ± 2.05%, error bar: standard deviation, P = 0.0087, n = 3 independent experiments). A frequency higher than 20 Hz potentially exceeds the step-out frequency at which mCNT rotation cannot respond fast enough to follow alteration of the actuation field, thereby resulting in less rotational energy delivered to cells and lower cell death rate (20 Hz vs. 30 Hz, 12.64 ± 2.05% vs. 7.70 ± 3.56%, error bar: standard deviation, P = 0.0186, n = 6 independent experiments, **Figure S1f**). Frequency of 20 Hz and magnitude of 20 mT were used in subsequent experiments.

The magnetic field used in treatment was in the range of 20 to 40 mT, with a magnetic gradient smaller than 2 T/m. For an iron particle clump with a diameter 16.25 ± 3.24 nm, the magnetic gradient force for pulling iron particle clump towards outside of carbon nanotube is smaller than 2.1×10^{-15} N. TEM with EDX before and

after magnetic field actuation showed that there was no significant difference in iron content within carbon nanotubes, indicating that the applied magnetic field actuation did not cause iron content loss.

The size and iron concentration can affect the motility performance of mCNTs. Because of the larger moment of inertia and larger magnetic moment, a longer size and higher iron concentration of mCNTs can potentially result in larger mechanical work delivered to the cell under the same magnetic field. However, a longer size and higher iron concentration could also change the frequency response of the mCNTs and/or lower the amount of mCNTs internalized by the cell. Future work to optimize mCNT length and iron concentration may further enhance treatment efficacy.

III. Magnetic field generation for *in vivo* treatment

Mechanical work exerted by mCNTs may cause cell death in the healthy region surrounding the tumor region. Hence, our treatment field was designed to maintain sufficient rotational energy within tumor while minimizing rotational energy outside the tumor region. The techniques include coordinate transformation from stereotaxic coordinates to Cartesian coordinates (through top-down and side view of the bioluminescence images), and magnetic field modeling for confining the region with a rotating magnetic field. In the magnetic field treatment system, the position of each magnetic pole was captured by camera mounted on the system with coordinates within the Cartesian coordinate system; when a mouse was placed within the workspace between each of the magnetic poles, the Cartesian coordinate of mouse's eyes and nose tip were also captured by the camera (**Figure 2f**). The location of tumor center (i.e., Cartesian coordinate) was calculated through its relative position to mouse's eyes and nose top based on top-down and side view of the bioluminescent image (**Figure 2g**). Based on the location of tumor center and tumor size, the magnetic field model was used to generate a rotating magnetic field within tumor while minimizing field strength in brain region surrounding the tumor.

In the magnetic field control strategy (**Figure S7**), a rotating magnetic field with a field strength maintained at 20 mT was applied to tumor region to induce tumor cell death, while the magnetic field strength was lower outside the tumor region. In the first quarter of a control cycle (0-1/4T), a pair of adjacent coils function as dominant coils for generating the rotating magnetic field. Meanwhile, the other pair of coils act as auxiliary coils to attenuate the magnetic field outside the target region. In the subsequent quarter of the same cycle (1/4T-1/2T), dominant coils and auxiliary coils are shifted clockwise by one coil. In this quarter, the magnetic field distribution changes, and the non-tumor region treated in the previous quarter, is now located in a region with low magnetic field strength. Dominant coils shift four times to complete a full control cycle T. Throughout a control cycle, the magnetic field in target region is always sufficiently large, maintaining strong treatment effects. However, outside the target region, the non-tumor region is periodically (a quarter of a cycle) subjected

to high and low magnetic field strength (**Figure S7b**). As such, only the specified tumor region is subjected to strong rotating field strength throughout the treatment cycle, whereas the non-tumor region is only subjected to a quarter of the cycle.

IV. Finite element simulation for estimating rotational energy delivered

A computational model was developed using COMSOL Multiphysics 5.5 to simulate magnetic field strength distribution within the workspace. The model consisted of four identical coils, each with an outer radius of 32.5 mm, an inner radius of 18 mm and a thickness of 22 mm. An iron core with a radius of 18 mm was in each coil. The midpoint between mouse's eyes was positioned at the center of workspace. The size and location of tumor region were determined based on bioluminescence images, with the radius set as 2.8 mm and centered at (1.5 mm, 4.5 mm). The default mesh size was 2.4 mm.

A spherical magnetic insulation was imposed to enclose the model, which set the tangential components of the magnetic potential to zero at the boundary. In the model, each coil was wired with Gauge 16 copper wires with a conductivity of 6×10^7 S/m, and the number of turns was set as 900 as in our experimental system. The material of iron core was assigned to be 4140 steel with a relative permeability of 700. A series of sinusoidal electrical current sequences, as determined by our magnetic field control strategy, were supplied to coils to generate rotational magnetic field. The maximum amplitude of electrical current was set at 5 A based on hardware limitation of our custom-designed current amplifier. The current waveform supplied to each coil was shown in **Figure S7a**. Three mesh sizes were tested. "Fine", "Finer" and "Extra Fine" (defined by COMSOL) meshes with a mesh size of 2.4 mm, 0.96 mm, and 0.36 mm, respectively, were used for comparison. The maximum differences between three computational meshes were less than 1% for magnetic field strength within the workspace.

In simulation, in a tumor region of 2.8 mm in radius and mCNTs of 0.48 µm in length and 52 wt% in iron percentage, the designed target magnetic field delivers a total of 2.5×10^{-16} J rotational energy to tumor during one-hour treatment, compared with 2.55×10^{-16} J under a uniform rotating magnetic field of 20 mT field strength. In the tissue surrounding the tumor, the rotational energy delivered was limited to 0.16×10^{-16} J (**Figure S7**).

V. Mechanical environment and treatment efficacy

The *in vitro* and *in vivo* mechanical environments are different. For instance, plastic petri dish has a stiffness about 2 GPa, and brain tumor tissue has a stiffness ranging from 500 to 10,000 Pa *in vivo (69)*. To study whether microenvironmental stiffness difference can cause variations in treatment efficacy, we cultured GBM

cells on substrates with different mechanical stiffness (200, 500, 1,000 Pa) and treated the cells with mCNT and magnetic field. The results (**Figure S18**) showed significant increase in treatment efficacy with the decrease of substrate stiffness.

Supplementary Figures

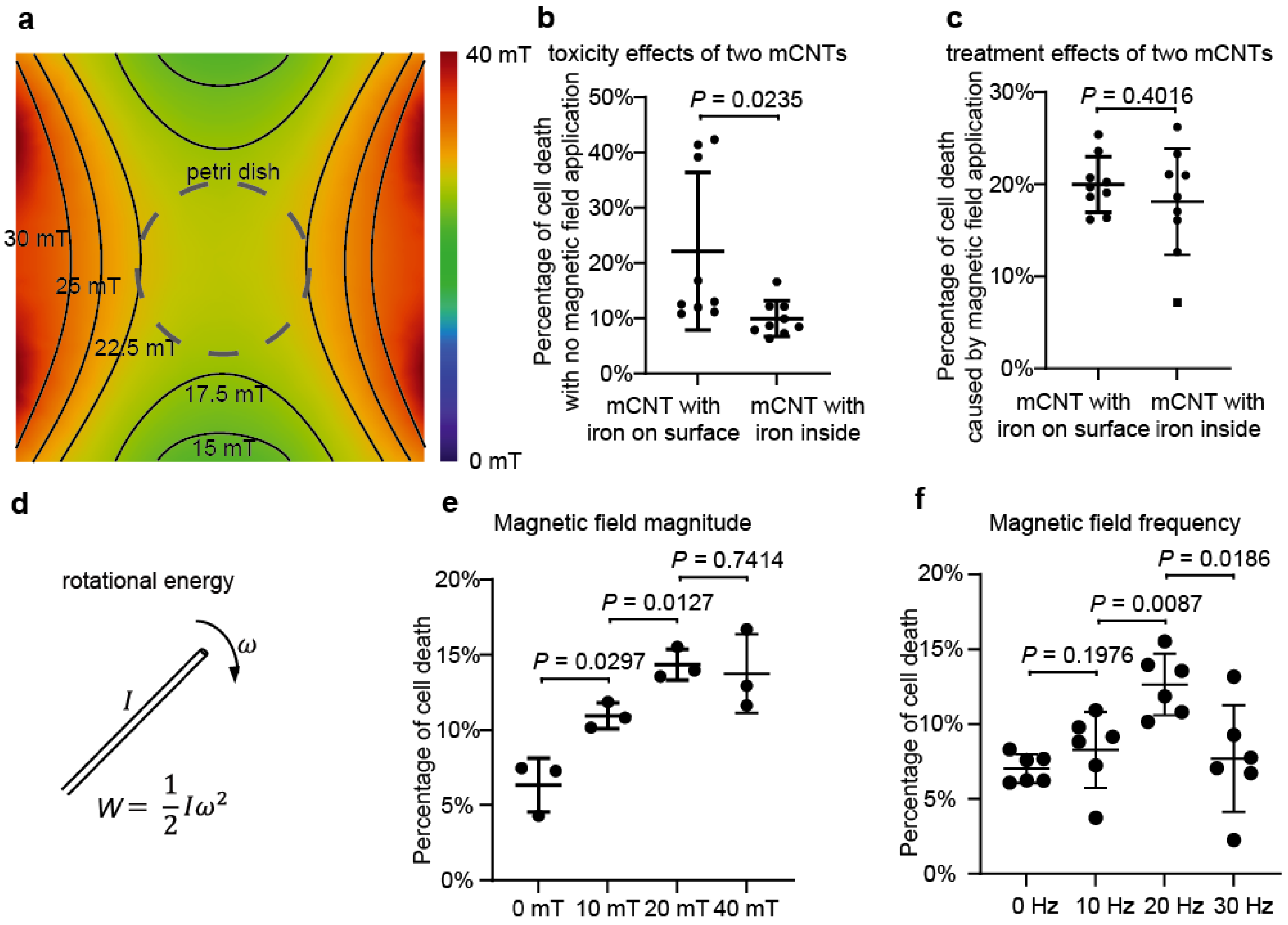

Figure S1 Treatment parameters (mCNT type, magnetic field magnitude, magnetic field frequency) for mCNT + magnetic treatment. (a) Magnetic field generated for *in vitro* treatment. The magnetic field strength within petri dish is 20 ± 2 mT. (b) Toxicity effects for two types of mCNTs, quantified as the percentage of cell death with no magnetic field treatment. n = 9 independent experiments, error bar: standard deviations. (c) Treatment effect of two types of mCNTs, quantified as percentage of cell death caused by magnetic field treatment. n = 9 independent experiments, error bar: standard deviations. (d) Schematic of mCNT rotation generates rotational energy. (e) Cell death rate with different magnetic field magnitude. n = 3 independent experiments, error bar: standard deviations. (f) Cell death rate with different magnetic field frequency. n = 3 independent experiments, error bar: standard deviations.

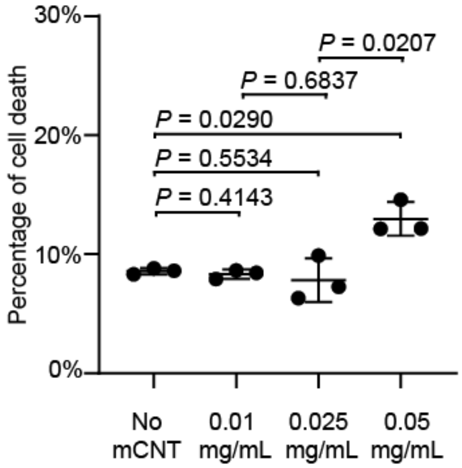

Figure S2. **Cell death rate of cells cultured with different concentrations of mCNTs for 24 hours without magnetic field applied.** Control group receives only PBS without mCNTs. *n* = 3 independent experiments. Error bar: standard deviation.

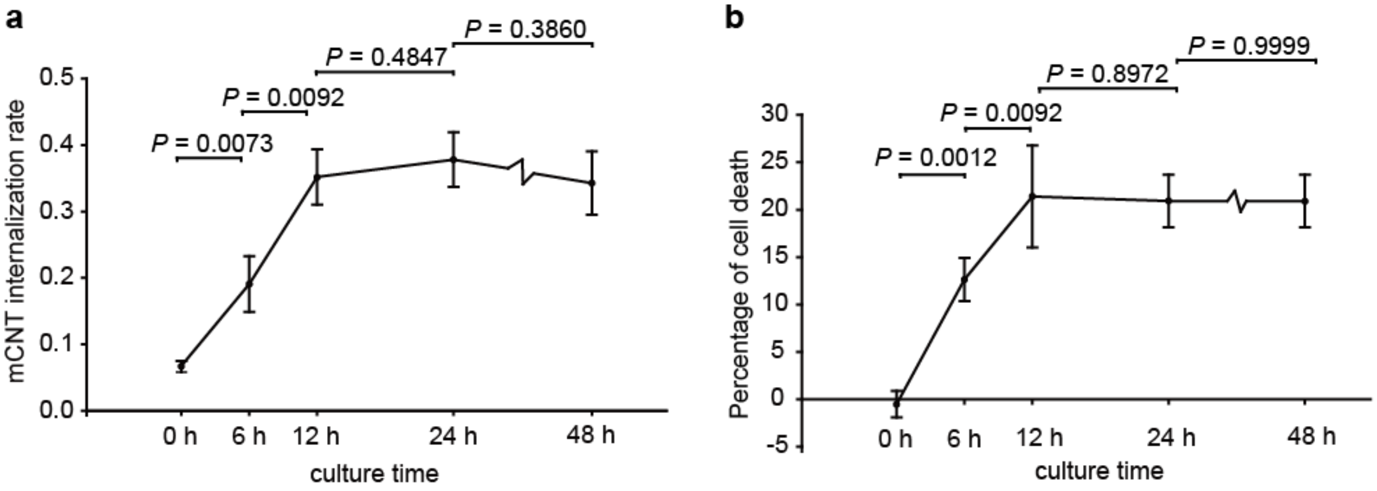

Figure S3. **Time window experiments for mCNT internalization and treatment efficacy.** (a) mCNT internalization rate after culturing mCNTs with G411 cells. mCNT internalization rate is calculated as mCNT area divided by cell area based on bright field microscopy images. *n* = 3 independent experiments. (b) Cell death rate after applying magnetic field treatment for G411 cells cultured with mCNTs. *n* = 3 independent experiments.

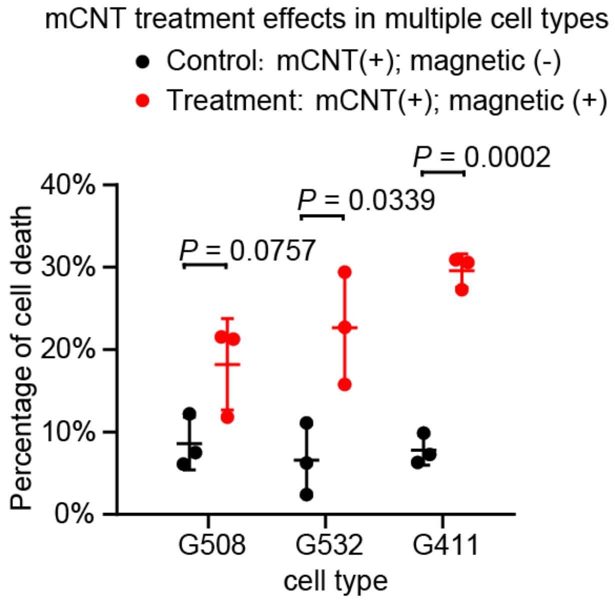

Figure S4. **Cell death rate after mCNT + magnetic field treatment in different GBM cell lines.** Control group receives mCNTs without magnetic field applied. n = 3 independent experiments, error bar: standard deviations.

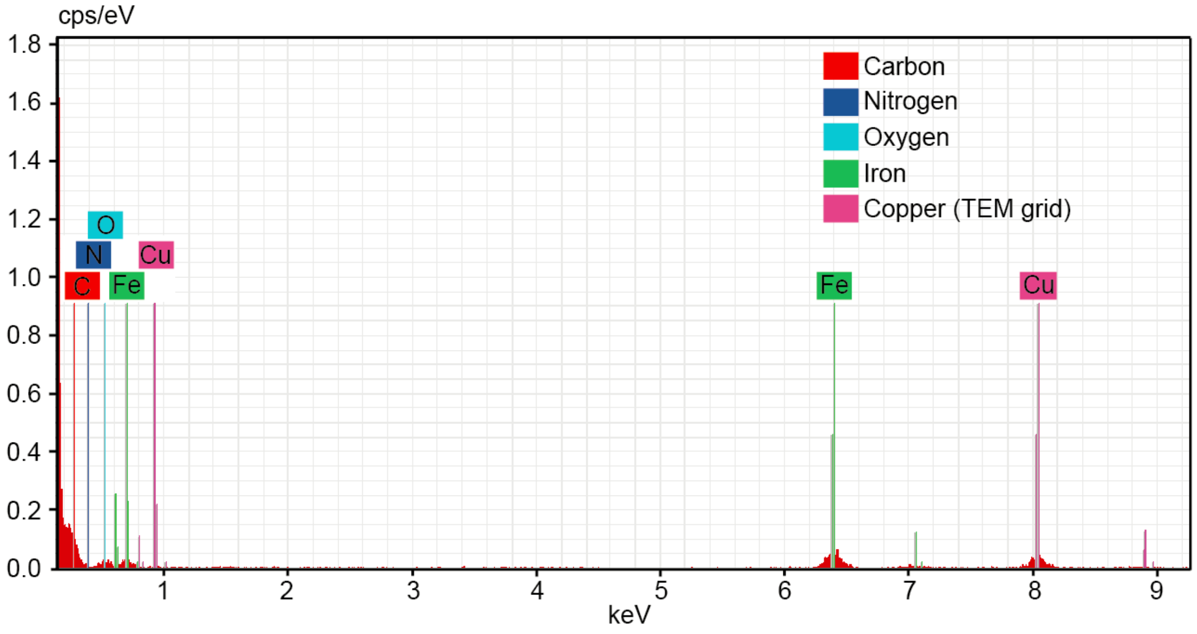

Figure S5. Energy dispersive X-ray spectroscopy (EDX) spectrum of mCNTs under TEM.

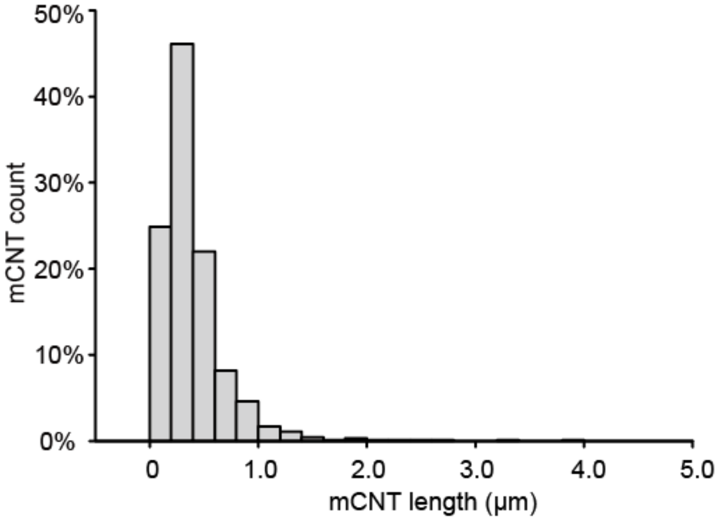

Figure S6. **The length of mCNTs distributed within a cell.** The length of mCNTs was quantified based on fluorescence images. *n* = 400 clusters of mCNTs.

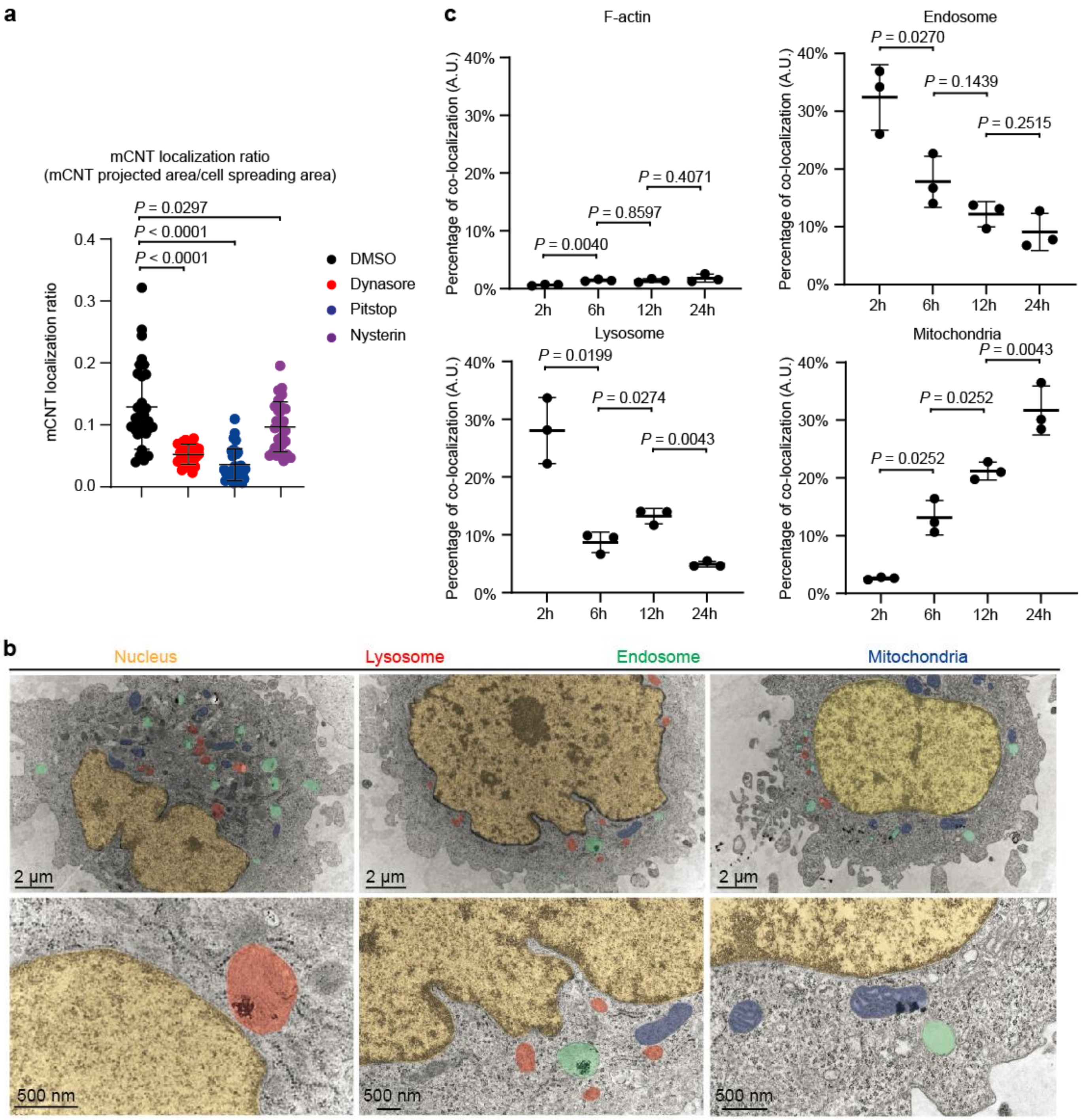

Figure S7 Entry route of mCNTs into GBM cells. (a) mCNT localization ratio after treating with endocytosis inhibitors. *n* = 30 cells, error bar: standard deviations. (b) TEM images of G411 GBM cells with mCNTs. The results showed mCNTs localized at lysosome, endosome, and mitochondria. Identification of intracellular organelles was based on “cell and organelles Dr. Jastrow’s electron microscopic atlas” *(68).* (c) Percentage of mCNT co-localization with F-actin, endosome, lysosome, and mitochondria, after co-culturing for 2, 6, 12, and 24 hours. The percentage was calculated using mCNT fluorescent signal overlapping with fluorescent signal from intracellular organelles divided by total mCNT fluorescent signal in each image. *n* = 3 images, error bar: standard deviations.

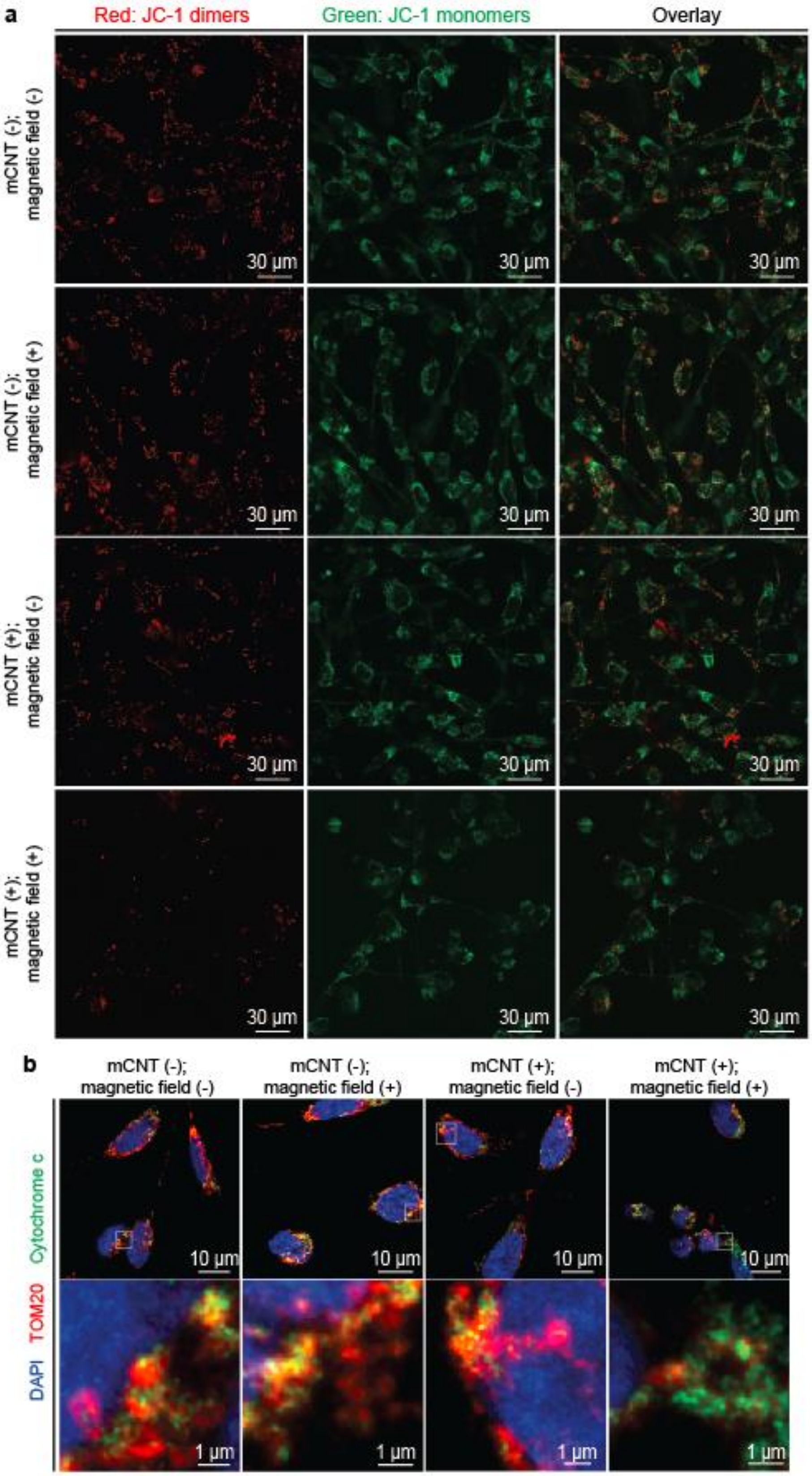

Figure S8. **mCNT treatment effects on mitochondria membrane potential and cytochrome C release.** (a) JC-1 staining shows depolarization of mitochondrial membrane potential induced by mCNT + magnetic treatment. (b) Immunocytochemical analysis demonstrates cytochrome c release into cytosol after mCNT + magnetic treatment.

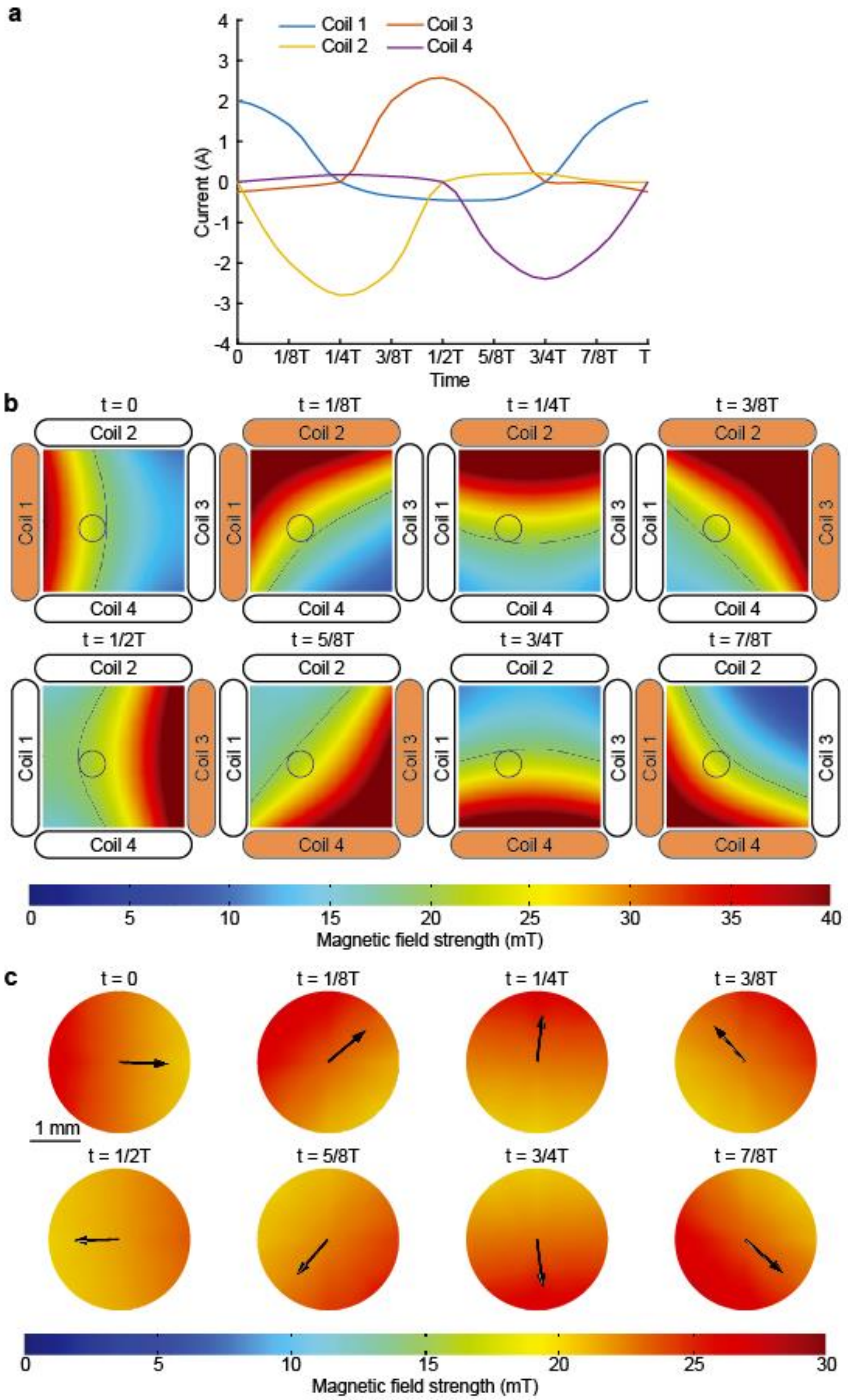

Figure S9. **Finite element analysis of the magnetic field within the workspace.** (a) Current waveform supplied to each coil for generating a targeted rotating magnetic field within tumor region. (b) and (c) Finite element simulation of magnetic field generated within the workspace. Circle indicates tumor region. (c) In tumor region, the strategy maintains magnetic field strength at ~20 mT.

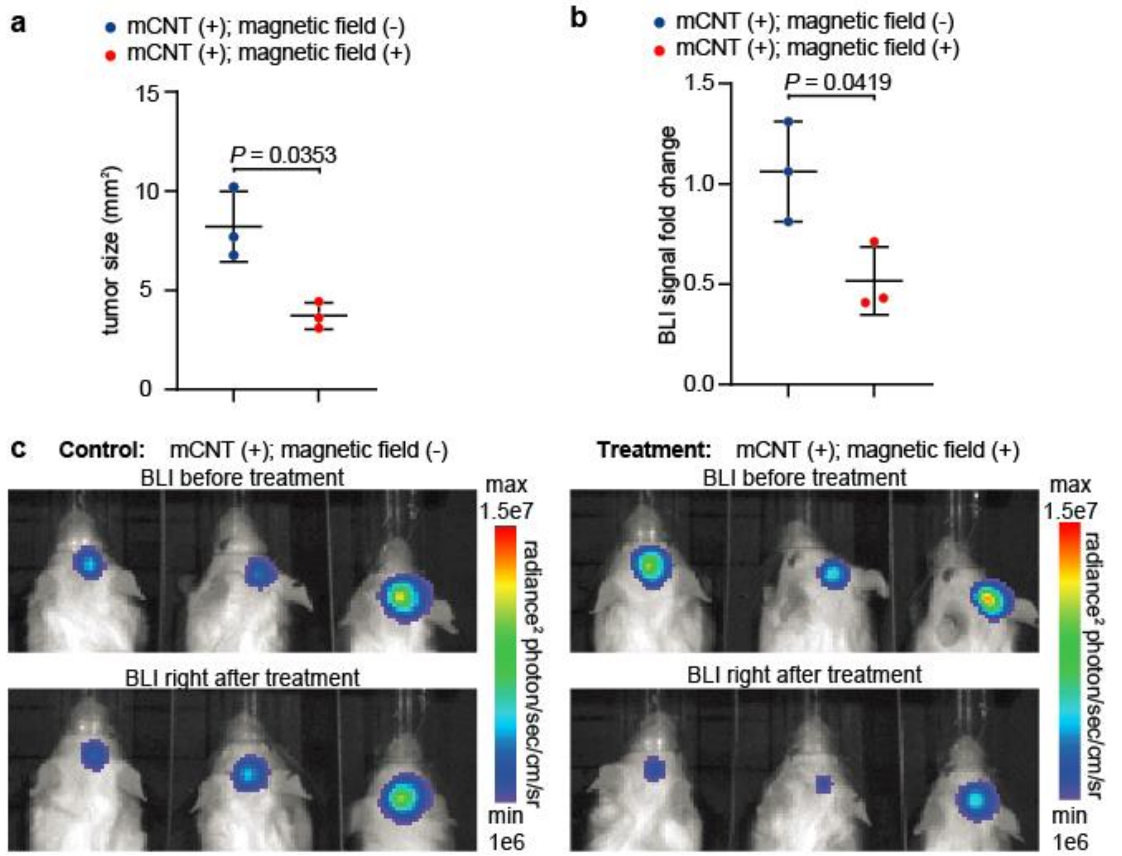

Figure S10 Tumor area and BLI signal before and after mCNT treatment. (a) Tumor area calculated from H&E staining after the 5th treatment, *n* = 3 independently treated mice, error bar: standard deviation. Representative H&E staining images are shown in **Figure 3c**. (b) Fold changes of BLI signals immediately after magnetic treatment. *n* = 3 mice, error bar: standard deviations. (c) BLI images of GBM-bearing mice before magnetic treatment and immediately after magnetic treatment. The images were taken immediately after the 3rd treatment (i.e., 13 days post tumor implantation). Control group only received anesthesia for 30 mins. Mouse model: G411 xenograft model.

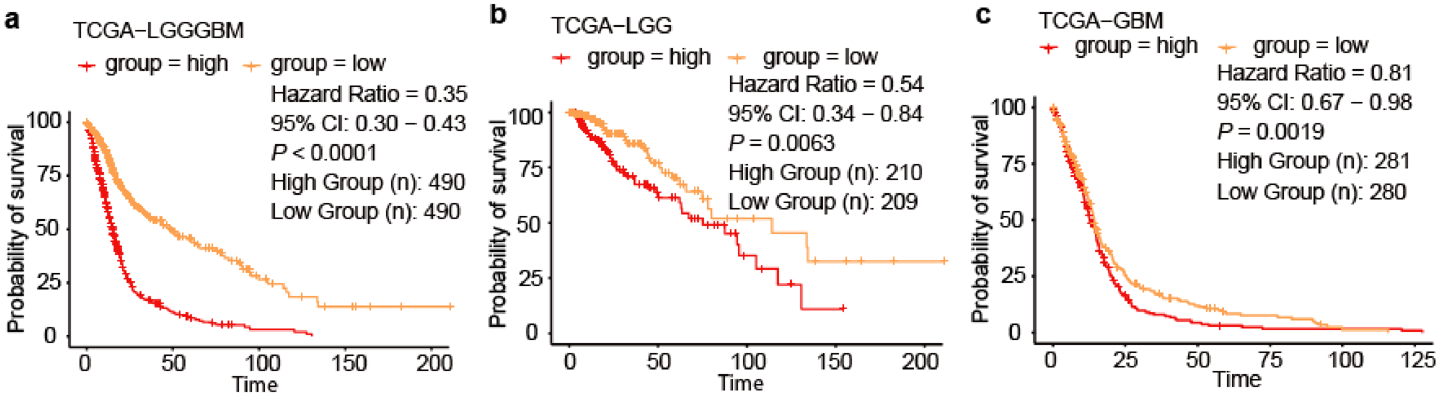

Figure S11. **High *CD44* expression is associated with shorter survival of glioma patients.** (a-c) Kaplan-Meier analysis shows that patients with high *CD44* expression in (a) TCGA-LGG/GBM, (b) TCGA-LGG, and (c) TCGA-GBM datasets exhibit worse overall survival than patients with low *CD44* expression.

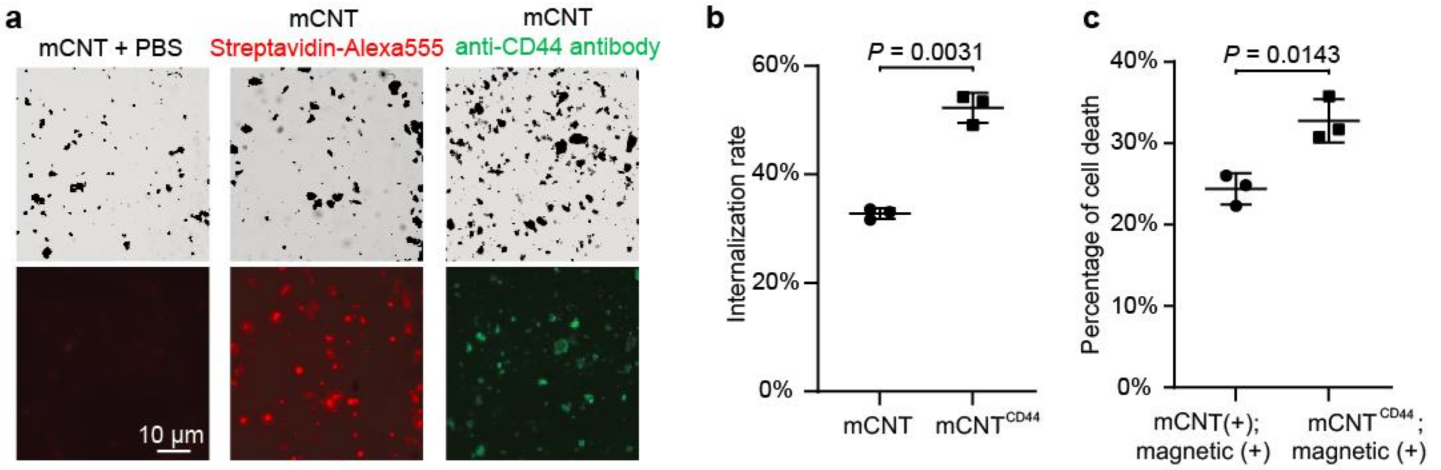

Figure S12. **Internalization and treatment efficacy comparison of functionalized mCNT and un-functionalized mCNT.** (a) Functionalization of mCNTs is validated by Streptavidin-Alexa 555 and anti-mouse Alexa 488 secondary antibody. (b) Internalization of mCNT and mCNT^{CD44} in G411 cells. Internalization rate is defined as the area of mCNT or mCNT^{CD44} over cell spreading area, after culturing mCNT or mCNT^{CD44} with cells for 24 hours. n = 3 independent experiments. Error bar: standard deviation. (c) The percentage of cell death rate *in vitro* on G411 caused by mCNT or mCNT^{CD44}. n = 3 independent experiments. Error bar: standard deviation.

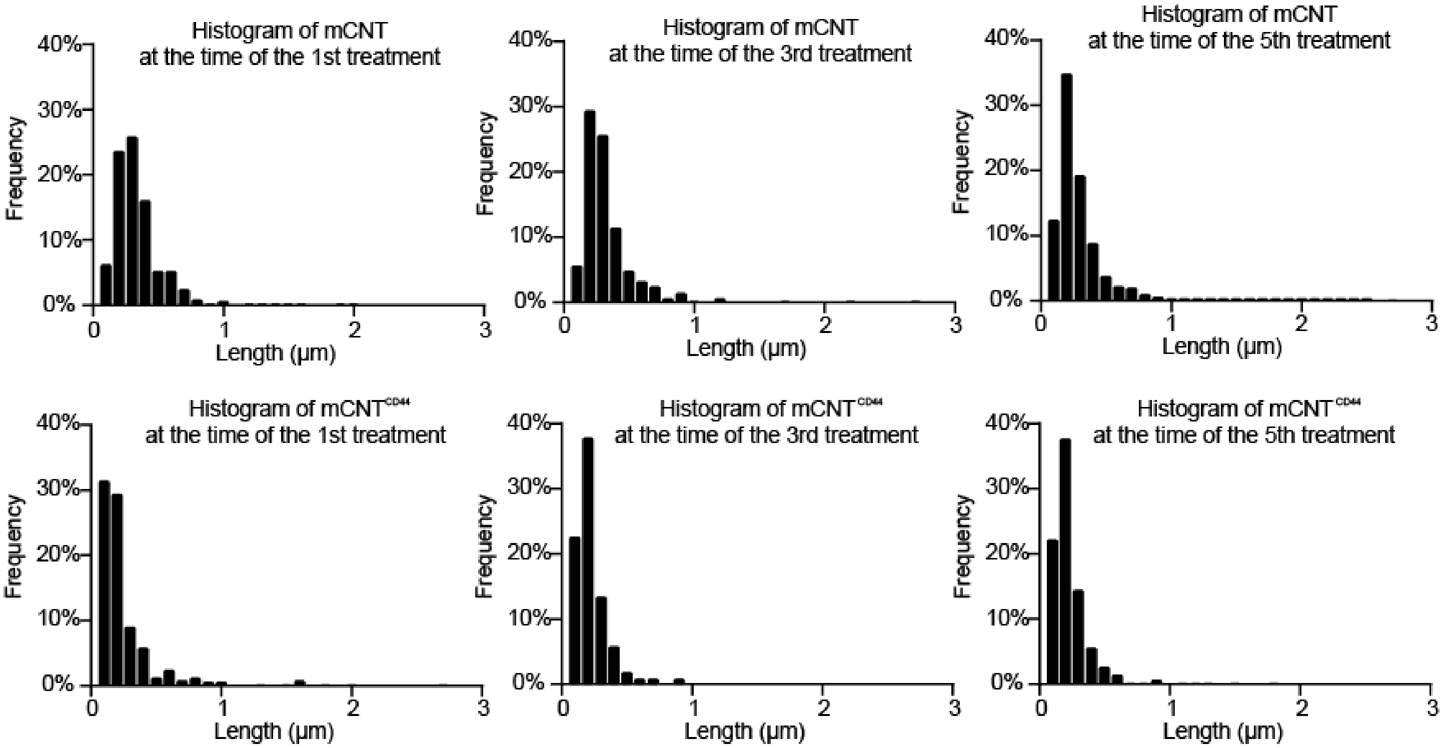

Figure S13 Size distribution of mCNT and mCNT^{CD44} aggregates *in vivo* after 1st, 3rd, 5th treatment.

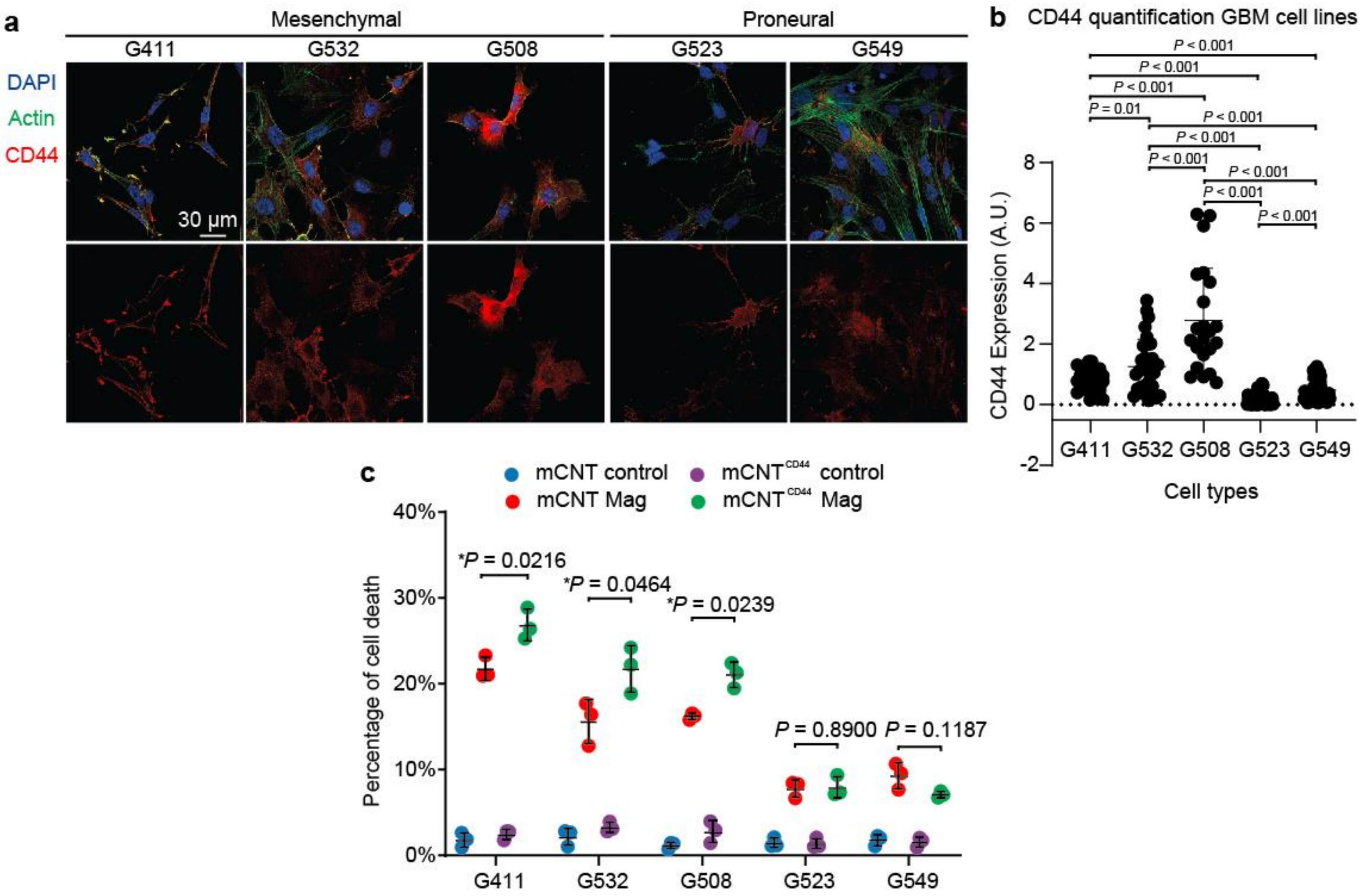

Figure S14. CD44 expression and mCNT^{CD44} treatment efficacy in GBM cell lines. (a) Immunostaining of CD44 in GBM cell lines of mesenchymal subtype (G411, G532, G508) and proneural subtype (G523, G549). (b) Quantification of CD44 expression in G411, G532, G508, G523, and G549. n = 40 cells, error bar: standard deviation. (c) Treatment effects (i.e., percentage of cell death) using mCNT and mCNT^{CD44} on various GBM cell lines. n = 3 independently repeated experiments, error bar: standard deviation.

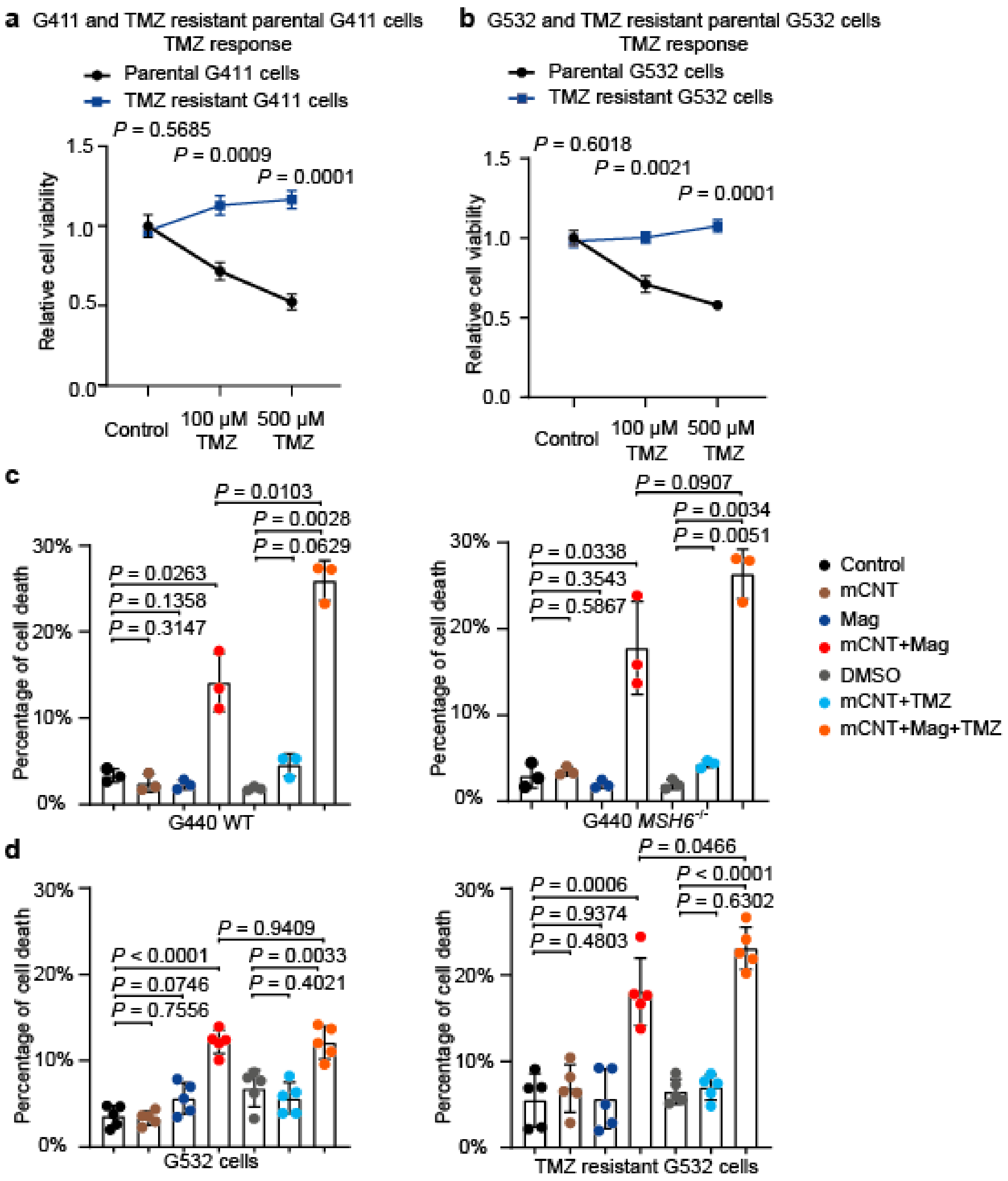

Figure S15. **mCNT treatment is effective against TMZ resistant GBM cell lines *in vitro*.** (a) Relative cell viability after treating parental and TMZ-resistant G411 cells with DMSO (control), 100 µM TMZ, and 500 µM TMZ for 5 days. n = 3 independent experiments. Error bar: standard deviation. (b) Relative cell viability after treating parental and TMZ resistant G532 cells with DMSO (control), 100 µM TMZ, and 500 µM TMZ for 5 days. n = 3 independent experiments. Error bar: standard deviation. (c) Cell death rate for cells treated with mCNTs, magnetic field treatment, TMZ, and their combinations, for G440 wild type cells and G440 *MSH6*$^{-/-}$ cells. n = 3 independent experiments. Error bar: standard deviation. (d) Cell death rate for cells treated with mCNTs, magnetic field treatment, TMZ, and their combinations, for parental and TMZ resistant G532 cells. n = 3 independent experiments. Error bar: standard deviation.

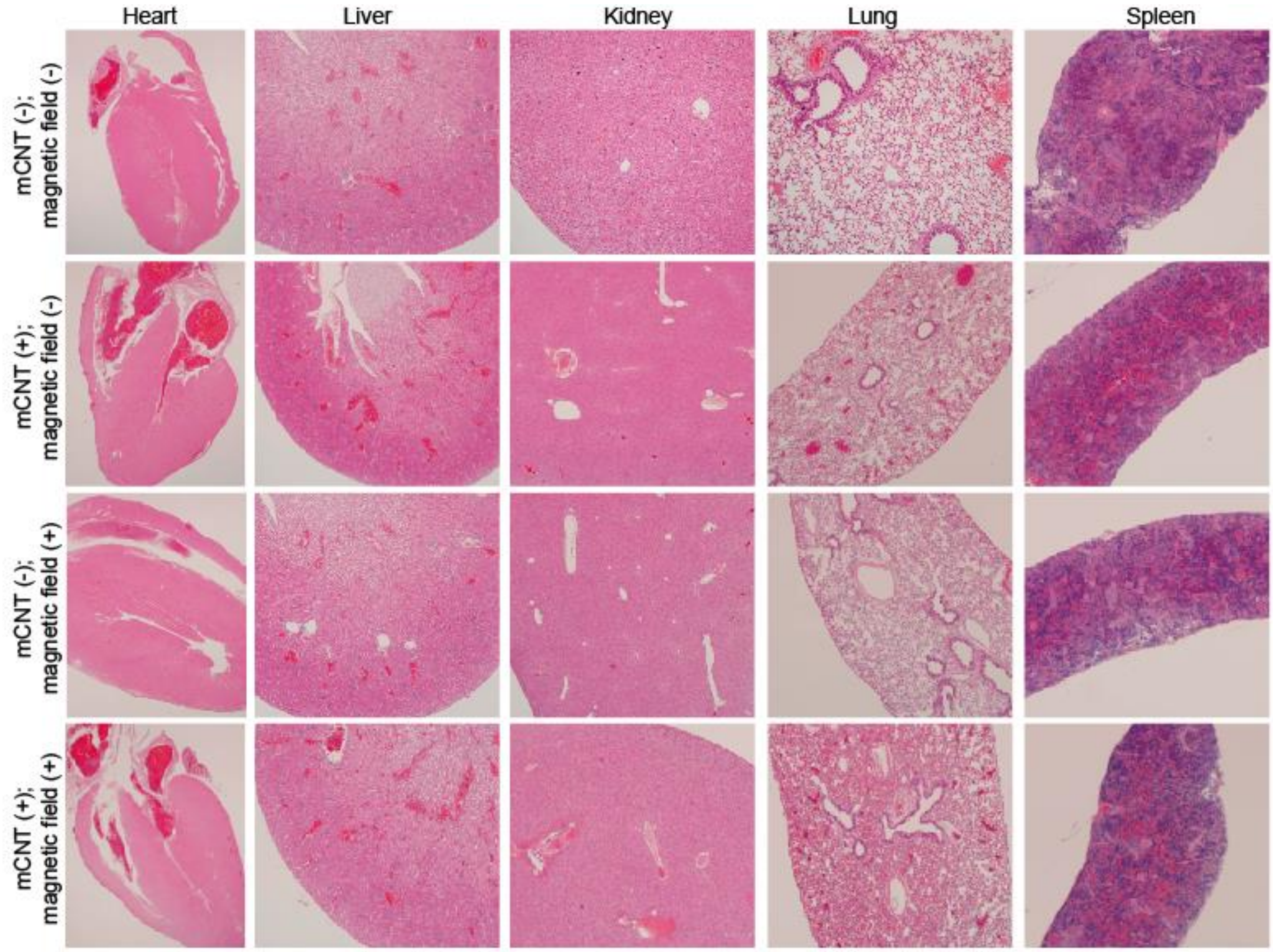

Figure S16. **Histological images of major organs.** Histological analyses show no overt pathological features in hearts, livers, kidneys, lungs, or spleens of mice treated with mCNT + magnetic field.

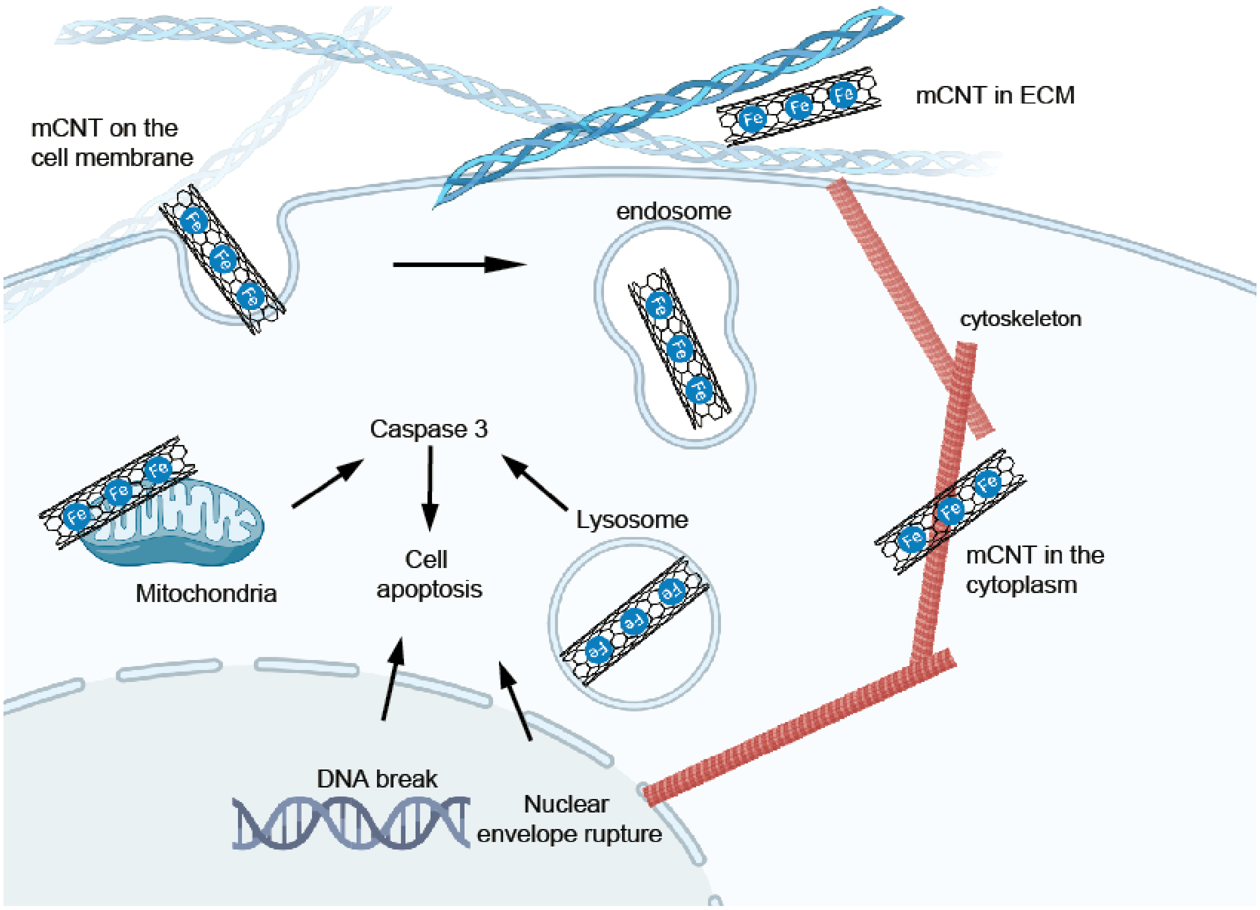

Figure S17. **Proposed mechanisms by which mCNT + magnetic field treatment induce GBM cell death.** mCNTs localize within the extracellular space, on cell membrane, and within the cell. We propose that mechanical torque from magnetic actuation can be transmitted from extracellular space to inside of the cell, generated directly on cell membrane, and generated within the cell to mechanically damage intracellular organelles, increase DNA damage, and induce cell apoptosis.

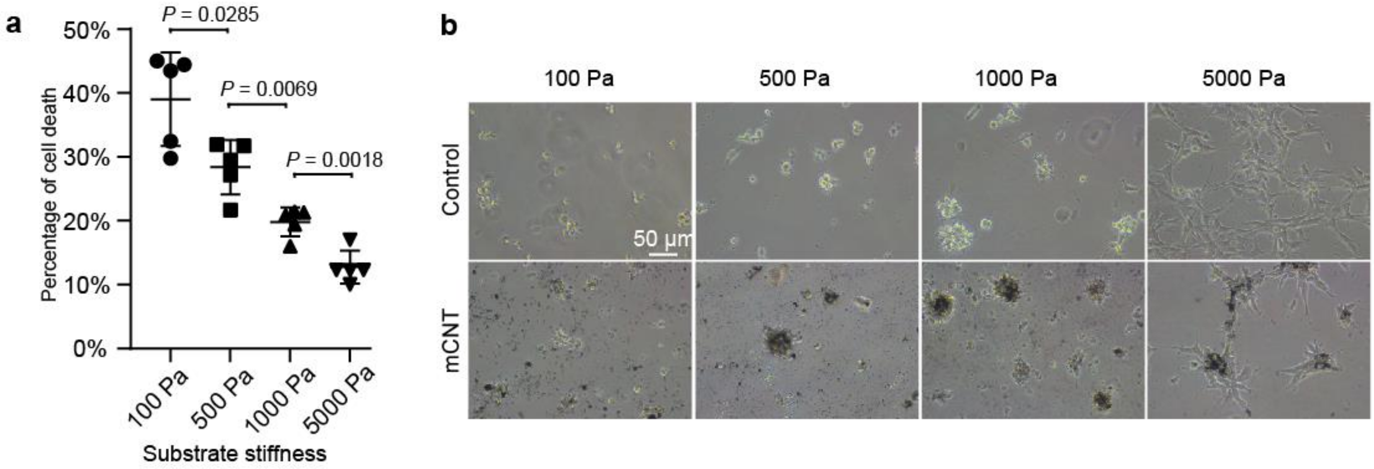

Figure S18. Substrate stiffness effects in treatment efficacy. (a) Percentage of cell death induced by mCNT + magnetic treatment for cells seeded on substrates with different stiffness. n = 5 independent experiments. Error bar: standard deviation. (b) Representative images of cells seeded on substrates with different stiffness.

Supplementary Video 1 mCNT actuation under magnetic field. The G411 cells were co-cultured with mCNT for 24 hours, then washed by PBS three times. After the extensive wash, the remaining mCNT aggregates were located on or across cell membrane and inside the cell. The video shows motion of a mCNT aggregate inside a cell under a rotating magnetic field (20 mT, 20 Hz).

REFERENCES AND NOTES

1. N. Grech, T. Dalli, S. Mizzi, L. Meilak, N. Calleja, A. Zrinzo, Rising incidence of glioblastoma multiforme in a well-defined population. *Cureus* **12**, e8195 2020.

2. R. Stupp, W. P. Mason, M. J. van den Bent, M. Weller, B. Fisher, M. J. Taphoorn, K. Belanger, A. A. Brandes, C. Marosi, U. Bogdahn, J. Curschmann, R. C. Janzer, S. K. Ludwin, T. Gorlia, A. Allgeier, D. Lacombe, J G. Cairncross, E. Eisenhauer, R. O. Mirimanoff; European Organisation for Research and Treatment of Cancer Brain Tumor and Radiotherapy Groups; National Cancer Institute of Canada Clinical Trials Group, Radiotherapy plus concomitant and adjuvant temozolomide for glioblastoma. *N. Engl. J. Med.* **352**, 987–996 (2005).

3. S. Y. Lee, Temozolomide resistance in glioblastoma multiforme. *Genes Dis.* **3**, 198–210 (2016)

4. P. Zhu, X. L. Du, G. Lu, J.-J. Zhu, Survival benefit of glioblastoma patients after FDA approval of temozolomide concomitant with radiation and bevacizumab: A population-based study. *Oncotarget* **8**, 44015–44031 (2017).

5. M. H. Cohen, J. R. Johnson, R. Pazdur, Food and drug administration drug approval summary: Temozolomide plus radiation therapy for the treatment of newly diagnosed glioblastoma multiforme. *Clin. Cancer Res.* **11**, 6767–6771 (2005).

6. S. Jiapaer, T. Furuta, S. Tanaka, T. Kitabayashi, M. Nakada, Potential strategies overcoming the temozolomide resistance for glioblastoma. *Neurol. Med. Chir. (Tokyo)* **58**, 405–421 (2018).

7. J. R. D. Pearson, T. Regad, Targeting cellular pathways in glioblastoma multiforme. *Signal Transduct. Target. Ther.* **2**, 17040 (2017).

8. D. Cai, J. M. Mataraza, Z.H. Qin, Z. Huang, J. Huang, T. C. Chiles, D. Carnahan, K. Kempa, Z. Ren, Highly efficient molecular delivery into mammalian cells using carbon nanotube spearing. *Nat. Methods* **2**, 449–454 (2005).

9. W. Shao, A. Paul, B. Zhao, C. Lee, L. Rodes, S. Prakash, Carbon nanotube lipid drug approach for targeted delivery of a chemotherapy drug in a human breast cancer xenograft animal model. *Biomaterials* **34**, 10109–10119 (2013).

10. A. A. Bhirde, V. Patel, J. Gavard, G. Zhang, A. A. Sousa, A. Masedunskas, R. D. Leapman, R. Weigert, J. S. Gutkind, J. F. Rusling, Targeted killing of cancer cells in vivo and in vitro with EGF-directed carbon nanotube-based drug delivery. *ACS Nano* **3**, 307–316 (2009).

11. J.-W. Kim, E. I. Galanzha, E. V. Shashkov, H.M. Moon, V. P. Zharov, Golden carbon nanotubes as multimodal photoacoustic and photothermal high-contrast molecular agents. *Nat. Nanotechnol.* **4**, 688–694 (2009).

12. K. Welsher, Z. Liu, S. P. Sherlock, J. T. Robinson, Z. Chen, D. Daranciang, H. Dai, A route to brightly fluorescent carbon nanotubes for near-infrared imaging in mice. *Nat. Nanotechnol.* **4**, 773–780 (2009).

13. B. Sitharaman, K. R. Kissell, K. B. Hartman, L. A. Tran, A. Baikalov, I. Rusakova, Y. Sun, H. A. Khant, S. J. Ludtke, W. Chiu, S. Laus, E. Tóth, L. Helm, A. E. Merbach, L. J. Wilson, Superparamagnetic gadonanotubes are high-performance MRI contrast agents. *Chem. Commun. (Camb)* **31**, 3915–3917 (2005).

14. H. Gong, R. Peng, Z. Liu, Carbon nanotubes for biomedical imaging: The recent advances. *Adv. Drug Deliv. Rev.* **65**, 1951–1963 (2013).

15. S. L. Edwards, J. A. Werkmeister, J. A. M. Ramshaw, Carbon nanotubes in scaffolds for tissue engineering. *Expert Rev. Med. Devices* **6**, 499–505 (2009).

16. J. Shi, P. W. Kantoff, R. Wooster, O. C. Farokhzad, Cancer nanomedicine: Progress, challenges and opportunities. *Nat. Rev. Cancer* **17**, 20–37 (2017).

17. R. van der Meel, E. Sulheim, Y. Shi, F. Kiessling, W. J. M. Mulder, T. Lammers, Smart cancer nanomedicine. *Nat. Nanotechnol.* **14**, 1007–1017 (2019).

18. X. Chen, S. Wanggou, A. Bodalia, M. Zhu, W. Dong, J. J. Fan, W. C. Yin, H.-K. Min, M. Hu, D. Draghici, W. Dou, F. Li, F. J. Coutinho, H. Whetstone, M. M. Kushida, P. B. Dirks, Y. Song, C.-C. Hui, Y. Sun, L.-Y. Wang, X. Li, X. Huang, A feedforward mechanism mediated by mechanosensitive ion channel PIEZO1 and tissue mechanics promotes glioma aggression. *Neuron* **100**, 799–815.e7 (2018).

19. D.-H. Kim, E. A. Rozhkova, I. V. Ulasov, S. D. Bader, T. Rajh, M. S. Lesniak, V. Novosad, Biofunctionalized magnetic-vortex microdiscs for targeted cancer-cell destruction. *Nat. Mater.* **9**, 165–171 (2010).

20. D. Liu, L. Wang, Z. Wang, A. Cuschieri, Magnetoporation and magnetolysis of cancer cells via carbon nanotubes induced by rotating magnetic fields. *Nano Lett.* **12**, 5117–5121 (2012).

21. Y. Cheng, M. E. Muroski, D. C. M. C. Petit, R. Mansell, T. Vemulkar, R. A. Morshed, Y. Han, I. V. Balyasnikova, C. M. Horbinski, X. Huang, L. Zhang, R. P. Cowburn, M. S. Lesniak, Rotating magnetic field induced oscillation of magnetic particles for in vivo mechanical destruction of malignant glioma. *J. Control. Release* **223**, 75–84 (2016).

22. M. Chen, J. Wu, P. Ning, J. Wang, Z. Ma, L. Huang, G. R. Plaza, Y. Shen, C. Xu, Y. Han, M. S. Lesniak, Z. Liu, Y. Cheng, Remote control of mechanical forces via mitochondrial-targeted magnetic nanospinners for efficient cancer treatment. *Small* **16**, e1905424 (2020).

23. B. C. Prager, S. Bhargava, V. Mahadev, C. G. Hubert, J. N. Rich, Glioblastoma stem cells: Driving resilience through chaos. *Trends Cancer* **6**, 223–235 (2020).

24. S. Osuka, E. G. Van Meir, Overcoming therapeutic resistance in glioblastoma: The way forward. *J. Clin. Invest.* **127**, 415–426 (2017).

25. M. Samadishadlou, M. Farshbaf, N. Annabi, T. Kavetskyy, R. Khalilov, S. Saghfi, A. Akbarzadeh, S. Mousavi, Magnetic carbon nanotubes: Preparation, physical properties, and applications in biomedicine. *Artif. Cells Nanomed. biotechnol.*, **46**, 1314–1330 (2018).

26. S. Kumari, S. MG, S. Mayor, Endocytosis unplugged: Multiple ways to enter the cell. *Cell Res.* **20**, 256–275 (2010).

27. M. S. Robinson, C. Watts, M. Zerial, Membrane dynamics in endocytosis. *Cell* **84**, 13–21 (1996).

28. H.-Y. Yue, E. Bieberich, J. Xu, Promotion of endocytosis efficiency through an ATP-independent mechanism at rat calyx of Held terminals. *J. Physiol.* **595**, 5265–5284 (2017).

29. N. W. S. Kam, Z. Liu, H. Dai Carbon nanotubes as intracellular transporters for proteins and DNA: An investigation of the uptake mechanism and pathway. *Angew. Chem. Int. Ed. Engl.* **45**, 577–581 (2006).

30. D. Pantarotto, J.-P. Briand, M. Prato, A. Bianco, Translocation of bioactive peptides across cell membranes by carbon nanotubes. *Chem. Commun. (Camb)* **1**, 16–17 (2004).

31. Z. Wei, W. Su, H. Lou, S. Duan, G. Chen, Trafficking pathway between plasma membrane and mitochondria via clathrin-mediated endocytosis. *J. Mol. Cell Biol.* **10**, 539–548 (2018).

32. S. Cerri, C. Milanese, P. G. Mastroberardino, Endocytic iron trafficking and mitochondria in Parkinson's disease. *Int. J. Biochem. Cell Biol.* **110**, 70–74 (2019).

33. R. J. Snyder, K. C. Verhein, H. L. Vellers, A. B. Burkholder, S. Garantziotis, S. R. Kleeberger, Multi-walled carbon nanotubes upregulate mitochondrial gene expression and trigger mitochondrial dysfunction in primary human bronchial epithelial cells. *Nanotoxicology* **13**, 1344–1361 (2019).

34. A. Das, S. Nag, A. B. Mason, M. M. Barroso, Endosome-mitochondria interactions are modulated by iron release from transferrin. *J. Cell Biol.* **214**, 831–845 (2016).

35. A. Hamdi, T. M. Roshan, T. M. Kahawita, A. B. Mason, A. D. Sheftel, P. Ponka, Erythroid cell mitochondria receive endosomal iron by a "kiss-and-run" mechanism. *Biochim. Biophys. Acta.* **1863**, 2859–2867 (2016).

36. E. Gottlieb, S. M. Armour, M. H. Harris, C. B. Thompson, Mitochondrial membrane potential regulates matrix configuration and cytochrome c release during apoptosis. *Cell Death Differ.* **10**, 709–717 (2003).

37. C. Garrido, L. Galluzzi, M. Brunet, P.E. Puig, C. Didelot, G. Kroemer, Mechanisms of cytochrome c release from mitochondria. *Cell Death Differ.* **13**, 1423–1433 (2006)

38. Y. Xia, I. L. Ivanovska, K. Zhu, L. Smith, J. Irianto, C. R. Pfeifer, C. M. Alvey, J. Ji, D. Liu, S. Cho, R. R. Bennett, A. J. Liu, R. A. Greenberg, D. E. Discher, Nuclear rupture at sites of high curvature compromises retention of DNA repair factors. *J. Cell Biol.* **217**, 3796–3808 (2018).

39. D. Si, F. Yin, J. Peng, G. Zhang, High expression of CD44 predicts a poor prognosis in glioblastomas. *Cancer Manag. Res.* **12**, 769–775 (2020).

40. K. L. Mooney, W. Choy, S. Sidhu, P. Pelargos, T. T. Bui, B. Voth, N. Barnette, I. Yang, The role of CD44 in glioblastoma multiforme. *J. Clin. Neurosci.* **34**, 1–5 (2016).

41. Y. Xu, I. Stamenkovic, Q. Yu, CD44 attenuates activation of the hippo signaling pathway and is a prime therapeutic target for glioblastoma. *Cancer Res.* **70**, 2455–2464 (2010).

42. M. J. Rahman, T. Mieno, Water-dispersible multiwalled carbon nanotubes obtained from citric-acid-assisted oxygen plasma functionalization. *J. Nanomater.* **2014**, 1–9 2014.

43. M. Yang, M. Zhang, Biodegradation of carbon nanotubes by macrophages. *Front. Mater.* **6**, 225 (2019).

44. C. Bussy, C. Hadad, M. Prato, A. Bianco, K. Kostarelos, Intracellular degradation of chemically functionalized carbon nanotubes using a long-term primary microglial culture model. *Nanoscale* **8**, 590–601 (2016).

45. H. Kafa, J. T.-W. Wang, N. Rubio, K. Venner, G. Anderson, E. Pach, B. Ballesteros, J. E. Preston, N. J. Abbott, K. T. Al-Jamal, The interaction of carbon nanotubes with an in vitro blood-brain barrier model and mouse brain in vivo. *Biomaterials* **53**, 437–452 (2015).

46. E. B. Postnikov, A. I. Lavrova, D. E. Postnov, Transport in the brain extracellular space: Diffusion, but which kind? *Int. J. Mol. Sci.* **23**, 12401 (2022).

47. Z. Liu, C. Davis, W. Cai, L. He, X. Chen, H. Dai, Circulation and long-term fate of functionalized, biocompatible single-walled carbon nanotubes in mice probed by Raman spectroscopy. *Proc. Natl. Acad. Sci. U.S.A.* **105**, 1410–1415 (2008).

48. A. Ruggiero, C. H. Villa, E. Bander, D. A. Rey, M. Bergkvist, C. A. Batt, K. Manova-Todorova, W. M. Deen, D. A. Scheinberg, M. R. McDevitt, Paradoxical glomerular filtration of carbon nanotubes. *Proc. Natl. Acad. Sci. U.S.A.* **107**, 12369–12374 (2010).

49. B. J. Denny, R. T. Wheelhouse, M. F. Stevens, L. L. Tsang, J. A. Slack, NMR and molecular modeling investigation of the mechanism of activation of the antitumor drug temozolomide and its interaction with DNA. *Biochemistry* **33**, 9045–9051 (1994).

50. G.-M. Li, Mechanisms and functions of DNA mismatch repair. *Cell Res.* **18**, 85–98 (2008).

51. S. Yip, J. Miao, D. P. Cahill, A. J. Iafrate, K. Aldape, C. L. Nutt, D. N. Louis, MSH6 mutations arise in glioblastomas during temozolomide therapy and mediate temozolomide resistance. *Clin. Cancer Res.* **15**, 4622–4629 (2009).

52. D. P. Cahill, K. K. Levine, R. A. Betensky, P. J. Codd, C. A. Romany, L. B. Reavie, T. T. Batchelor, P. A. Futreal, M. R. Stratton, W. T. Curry, A. J. Iafrate, D. N. Louis, Loss of the mismatch repair protein MSH6 in human glioblastomas is associated with tumor progression during temozolomide treatment. *Clin. Cancer Res.* **13**, 2038–2045 (2007).

53. F. Michor, M. A. Nowak, Y. Iwasa, Evolution of resistance to cancer therapy. *Curr. Pharm. Des.* **12**, 261–271 (2006).

54. H. S. Friedman, T. Kerby, H. Calvert, Temozolomide and treatment of malignant glioma. *Clin. Cancer Res.* **6**, 2585–2597 (2000).

55. N. Singh, A. Miner, L. Hennis, S. Mittal, Mechanisms of temozolomide resistance in glioblastoma–A comprehensive review. *Cancer Drug Resist.* **4**, 17–43 (2021).

56. H. F. van Thuijl, T. Mazor, B. E. Johnson, S. D. Fouse, K. Aihara, C. Hong, A. Malmström, M. Hallbeck, J. J. Heimans, J. J. Kloezeman, M. Stenmark-Askmalm, M. L. M. Lamfers, N.

Saito, H. Aburatani, A. Mukasa, M. S. Berger, P. Söderkvist, B. S. Taylor, A. M. Molinaro, P. Wesseling, J. C. Reijneveld, S. M. Chang, B. Ylstra, J. F. Costello, Evolution of DNA repair defects during malignant progression of low-grade gliomas after temozolomide treatment. *Acta Neuropathol.* **129**, 597–607 (2015).

57. B. E. Johnson, T. Mazor, C. Hong, M. Barnes, K. Aihara, C. Y. McLean, S. D. Fouse, S. Yamamoto, H. Ueda, K. Tatsuno, S. Asthana, L. E. Jalbert, S. J. Nelson, A. W. Bollen, W. C. Gustafson, E. Charron, W. A. Weiss, I. V. Smirnov, J. S. Song, A. B. Olshen, S. Cha, Y. Zhao, R. A. Moore, A. J. Mungall, S. J. M. Jones, M. Hirst, M. A. Marra, N. Saito, H. Aburatani, A. Mukasa, M. S. Berger, S. M. Chang, B. S. Taylor, J. F. Costello, Mutational analysis reveals the origin and therapy-driven evolution of recurrent glioma. *Science* **343**, 189–193 (2014).

58. R. Stupp, E. T. Wong, A. A. Kanner, D. Steinberg, H. Engelhard, V. Heidecke, E. D. Kirson, S. Taillibert, F. Liebermann, V. Dbalý, Z. Ram, J. L. Villano, N. Rainov, U. Weinberg, D. Schiff, L. Kunschner, J. Raizer, J. Honnorat, A. Sloan, M. Malkin, J. C. Landolfi, F. Payer, M. Mehdorn, R. J. Weil, S. C. Pannullo, M. Westphal, M. Smrcka, L. Chin, H. Kostron, S. Hofer, J. Bruce, R. Cosgrove, N. Paleologous, Y. Palti, P. H. Gutin, NovoTTF-100A versus physician's choice chemotherapy in recurrent glioblastoma: A randomised phase III trial of a novel treatment modality. *Eur. J. Cancer* **48**, 2192–2202 (2012).

59. R. Stupp, S. Taillibert, A. A. Kanner, S. Kesari, D. M. Steinberg, S. A. Toms, L. P. Taylor, F. Lieberman, A. Silvani, K. L. Fink, G. H. Barnett, J.-J. Zhu, J. W. Henson, H. H. Engelhard, T. C. Chen, D. D. Tran, J. Sroubek, N. D. Tran, A. F. Hottinger, J. Landolfi, R. Desai, M. Caroli, Y. Kew, J. Honnorat, A. Idbaih, E. D. Kirson, U. Weinberg, Y. Palti, M. E. Hegi, Z. Ram, Maintenance therapy with tumor-treating fields plus temozolomide vs temozolomide alone for glioblastoma: a randomized clinical trial. *JAMA* **314**, 2535–2543 (2015).

60. D. S. Baskin, M. A. Sharpe, L. Nguyen, S. A. Helekar, Case report: End-stage recurrent glioblastoma treated with a new noninvasive non-contact oncomagnetic device. *Front. Oncol.* **11**, 708017 (2021).

61. E. D. Kirson, V. Dbalý, F. Tovaryš, J. Vymazal, J. F. Soustiel, A. Itzhaki, D. Mordechovich, S. Steinberg-Shapira, Z. Gurvich, R. Schneiderman, Y. Wasserman, M. Salzberg, B. Ryffel, D. Goldsher, E. Dekel, Y. Palti, Alternating electric fields arrest cell proliferation in animal tumor models and human brain tumors. *Proc. Natl. Acad. Sci. U.S.A.* **104**, 10152–10157 (2007).

62. N. Gera, A. Yang, T. S. Holtzman, S. X. Lee, E. T. Wong, K. D. Swanson, Tumor treating fields perturb the localization of septins and cause aberrant mitotic exit. *PLOS ONE* **10**, e0125269 (2015).

63. J. Hu, S. Western, S. Kesari, Brainstem glioma in adults. *Front. Oncol.* **6**, 180 (2016).

64. G. MacLeod, D. A. Bozek, N. Rajakulendran, V. Monteiro, M. Ahmadi, Z. Steinhart, M. M. Kushida, H. Yu, F. J. Coutinho, F. M. G. Cavalli, I. Restall, X. Hao, T. Hart, H. A. Luchman, S. Weiss, P. B. Dirks, S. Angers, Genome-wide CRISPR-Cas9 screens expose genetic vulnerabilities and mechanisms of temozolomide sensitivity in glioblastoma stem cells. *Cell Rep.* **27**, 971–986.e9 (2019).

65. S. M. Pollard, K. Yoshikawa, I. D. Clarke, D. Danovi, S. Stricker, R. Russell, J. Bayani, R. Head, M. Lee, M. Bernstein, J. A. Squire, A. Smith, P. Dirks, Glioma stem cell lines expanded in adherent culture have tumor-specific phenotypes and are suitable for chemical and genetic screens. *Cell Stem Cell* **4**, 568–580 (2009).

66. F. Geng, H. Cong, Fe-filled carbon nanotube array with high coercivity. *Physica B. Condens. Matter* **382**, 300–304 (2006).

67. L. C. Crowley, B. J. Marfell, A. P. Scott, J. A. Boughaba, G. Chojnowski, M. E. Christensen, N. J. Waterhouse, Dead cert: Measuring cell death. *Cold Spring Harb. Protoc.* **2016**, pdb-top070318 (2016).

68. H. Jastrow, "Index of Dr. Jastrow's electron microscopic atlas," Dr. Jastrow's Electron Microscopic Atlas; www.drjastrow.de/WAI/EM/EMAtlas.html.

69. Y. A. Miroshnikova, J. K. Mouw, J. M. Barnes, M. W. Pickup, J. N. Lakins, Y. Kim, K. Lobo, A. I. Persson, G. F. Reis, T. R. McKnight, E. C. Holland, J. J. Phillips, V. M. Weaver, Tissue mechanics promote IDH1-dependent HIF1α–tenascin C feedback to regulate glioblastoma aggression. *Nat. Cell Biol.* **18**, 1336–1345 (2016).
